# The Beneficial Impact of Zinc Supplementation on the Vascular Tissue of the Abdominal Aorta under Repeated Intoxication with Cadmium: A Study in an In Vivo Experimental Model

**DOI:** 10.3390/nu14194080

**Published:** 2022-09-30

**Authors:** Małgorzata M. Brzóska, Magdalena Kozłowska, Joanna Rogalska

**Affiliations:** Department of Toxicology, Medical University of Bialystok, Adama Mickiewicza 2C Street, 15-222 Bialystok, Poland

**Keywords:** abdominal aorta, adhesive molecules, cadmium, endothelial cells, inflammation, oxidative stress, protection, serum, vascular tissue, zinc

## Abstract

In an in vivo rat model of human exposure to cadmium (Cd; 5 and 50 mg/L, 6 months), whether the supplementation with zinc (Zn; 30 and 60 mg/L, increasing its daily intake by 79% and 151%, respectively) protects against the unfavourable impact of this xenobiotic on the vascular tissue of the abdominal aorta was investigated. The treatment with Cd led to oxidative stress and increased the concentrations of pro-inflammatory interleukin 1β (IL-1β), total cholesterol (TC), triglycerides (TG), and endothelial nitric oxide synthase (eNOS) and decreased the concentration of anti-inflammatory interleukin 10 (IL-10) in the vascular tissue. Cd decreased the expression of intercellular adhesion molecule-1 (ICAM-1), platelet endothelial cell adhesion molecule-1 (PECAM-1), and L-selectin on the endothelial cells. The administration of Zn prevented most of the Cd-induced alterations or at least weakened them (except for the expression of adhesive molecules). In conclusion, Zn supplementation may protect from the toxic impact of Cd on the blood vessels and thus exert a beneficial influence on the cardiovascular system. The increase in the intake of Zn by 79% may be sufficient to provide this protection and the effect is related to the antioxidative, anti-inflammatory, and antiatherogenic properties of this essential element.

## 1. Introduction

Zinc (Zn) is an essential trace element involved in a variety of biological processes in the human organism, such as the growth, proliferation, and differentiation of cells among others [1,2,3]. The consumption of food products rich in Zn and supplementation with this element are widely recommended by nutritionists and health promoters [1,2,3]. The beneficial effects of Zn on the human body include support of the immune system, the treatment of the common cold, diarrhea, acne, and epilepsy [2,3]. This element can also be used in the management of the Coronavirus Disease 2019 [4]. Among numerous favourable effects of the supplementation with Zn, there are also experimental data on its protective impact against the toxic action of some heavy metals, including cadmium (Cd) [5,6,7,8,9,10,11].

The accumulation of Cd in the environment and outcomes of lifetime exposure to this xenobiotic are among the most disturbing ecological and health issues in industrialized countries [12,13,14,15,16,17,18,19,20]. Chronic, even low-level, intoxication with this trace element may lead to damage to the kidney, liver, and skeleton, as well as increase the risk of neurodegenerative diseases and cancer [12,13,15,16]. Moreover, it may result in severe cardiovascular pathologies (atherosclerosis, stroke, hypertension, and impaired heart function) and increase cardiovascular mortality [12,17,18,19,20,21]. The endothelial cells are considered to be the main target for the toxic action of Cd on the blood vessels [20,22,23,24]. Experimental data have revealed that this xenobiotic may be the cause of the dysfunction and death of the endothelial cells, followed by alterations in the endothelial integrity and vascular damage [20,22,23,24,25,26,27,28,29,30]. However, the mechanisms of the injury of the vessels due to the intoxication with Cd comparable to human exposure have not been studied yet. The global burden of cardiovascular diseases is constantly growing and is considered to be the main cause of death worldwide [31]. Thus, the unavoidable exposure to Cd [12,13] creates the need to recognize the risk of this xenobiotic-caused occurrence of these outcomes in the general population on the one hand and search for preventive strategies, on the other hand.

Taking into account the role of Zn in the functioning of the circulatory system [32,33,34,35] and the available data on its interactions with Cd [5,6,7,8,9,10,11,36,37], it was justifiable to suspect that the supplementation with this element may be one of the potential effective strategies against harmful cardiovascular outcomes of Cd. Zn and its associated transporter proteins are necessary for the proper integrity and function of both large vessels and microvessels [32,35]. This bioelement is an important vasodilator and essential component for the proper activity of *endothelial nitric oxide synthase* (eNOS) [32]. Zn has anti-inflammatory, antioxidative, and cytoprotective effects on the endothelium [25,32,35,38,39,40] and the latest data indicate that it may be a possible new agent in vascular therapy [32,33,34,35]. Moreover, it has been reported that Zn protects against Cd cytotoxicity in cultured vascular endothelial cells and bovine aorta endothelial cells [39,40]; nevertheless, there is a lack of data on the in vivo effect of its enhanced intake on the cardiovascular system under exposure to this xenobiotic. We have hypothesized that the potential protective effect of Zn may be related to its impact on the blood vessels. To investigate this hypothesis, we evaluated the most important biomarkers of oxidative stress (total antioxidative status—TAS, total oxidative status—TOS, and oxidative stress index—OSI) and inflammation (interleukin 1β—IL-1β and interleukin 10—IL-10), as well as other parameters influencing the functioning of the vessels (eNOS, total cholesterol—TC, and triglycerides—TG) in the abdominal aorta. Moreover, some indicators of inflammation and vascular endothelial growth factor (VEGF) were determined in the serum. The expression of adhesive molecules on the vascular endothelial cells and blood leukocytes was evaluated as well. The study was expected to provide new and practically useful data on the damaging influence of Cd on the blood vessels and evidence on the effectiveness of Zn supplementation in the protection from this xenobiotic-mediated injury of the cardiovascular system.

## 2. Materials and Methods

### 2.1. Experimental Animals

The experiment was conducted on 72 adult (10 weeks old) male Wistar rats (Hannover Wistar rats bred using the Charles River International Genetic Standardization Program—Crl: WI (Han)); certified Laboratory Animal House in Brwinów, Poland) kept under controlled conventional conditions (12-h light-dark cycle, temperature 22 ± 2 °C, relative humidity 50 ± 10%) with unrestricted access to standard rodent LSM dry chow (type of complete diet for rats and mice kept under laboratory conditions during the experiments) (Agropol, Motycz, Poland), containing 0.098 mg Cd/kg (determined in our laboratory [7]) and 48 mg Zn/kg (producer’s data) and drinking water (redistilled water free of contaminants: ≤0.05 μg Cd/L and ≤10 μg Zn/L [7]).

The research was approved by the Local Ethics Committee for Animal Experiments in Bialystok (Poland; approval No 2004/03 issued on 25 February 2004). All procedures involving animals were conducted according to the ethical principles, institutional guidelines, and the International Guide for the Use of Animals in Biomedical Research.

### 2.2. Exposure to Cd

Cd was administered in drinking water in the form of cadmium chloride (CdCl_2_ × 2½ H_2_O; POCh, Gliwice, Poland) at the concentration of 5 or 50 mg Cd/L. The aqueous solutions containing 5 and 50 mg Cd/L were made up by appropriate dilution, with redistilled water, of the solution of 1000 mg Cd/L (prepared by dissolving a certain amount of CdCl_2_ in redistilled water).

### 2.3. Supplementation with Zn

Zn was given to the rats in drinking water as zinc chloride (ZnCl_2_; Merck, Darmstadt, Germany) at the concentration of 30 or 60 mg Zn/L. The aqueous solutions containing 30 or 60 mg Zn/L were prepared by appropriate dilution, with redistilled water, of the solution of 1000 mg Zn/L (made up by dissolving a certain amount of ZnCl_2_ in redistilled water). In the case of Zn administration under the exposure to Cd, the solutions containing together Cd (5 or 50 mg/L) and Zn (30 or 60 mg/L) were prepared using the 1000 mg Cd/L and 1000 mg Zn/L solutions and redistilled water for their appropriate dilution.

### 2.4. Experimental Model

The rats were randomly assigned to the following experimental groups:

Control group: received drinking water without Cd and Zn addition;
Zn30 group: received Zn at the concentration of 30 mg/L of drinking water;
Zn60 group: received Zn at the concentration of 60 mg/L of drinking water;
Cd5 group: intoxicated with Cd at the concentration of 5 mg/L of drinking water;
Cd5 + Zn30 group: received drinking water containing 5 mg Cd/L and 30 mg Zn/L;
Cd5 + Zn60 group: received drinking water containing 5 mg Cd/L and 60 mg Zn/L;
Cd50 group: intoxicated with Cd at the concentration of 50 mg/L of drinking water;
Cd50 + Zn30 group: received drinking water containing 50 mg Cd/L and 30 mg Zn/L;
Cd50 + Zn60 group: received drinking water containing 50 mg Cd/L and 60 mg Zn/L.

There were 8 animals in each group. The investigation lasted 6 months. During the whole period of the experiment, all animals were maintained on the standard rodent LSM diet. The consumption of the diet was within the same range in all groups; therefore, the daily intakes of Cd and Zn with food were similar in all animals [7].

The daily consumption of Cd and Zn via drinking water was within the same range of values regardless of whether these elements were administered alone or in combination (Table 1) [7]. The mean daily intake of Cd by the rats treated with 5 and 50 mg Cd/L (alone and together with Zn) was 0.430 ± 0.019 mg/kg body weight (b.w.) (mean ± standard error (SE)) and 2.728 ± 0.198 mg/kg b.w., respectively [7].

The used levels of intoxication with Cd reflect moderate (5 mg Cd/L) and relatively high (50 mg Cd/L) exposure to this heavy metal in humans. Cd concentrations in the blood and urine (main markers of exposure to this toxic element) of the rats maintained on the water containing 5 mg Cd/L (1.001–2.064 μg/L and 0.010–0.019 μg/24 h, respectively; Supplementary Table S1) [7] are comparable with its concentrations nowadays noted in inhabitants of industrialized countries, including individuals exposed environmentally and/or occupationally and cigarette smokers (Supplementary Table S2). Although Cd concentrations in the blood and urine, comparable to these noted in the rats intoxicated with 50 mg Cd/L (13.81–17.25 μg/L and 0.155–0.335 μg/24 h, respectively; Supplementary Table S1) [7], are nowadays noted rarely in inhabitants of polluted areas or individuals occupationally exposed to Cd, they can still occur, especially in heavy tobacco smokers (Supplementary Table S2). For this purpose and to evaluate the dose–effect relationship, as well as to better investigate the possible mechanisms of the impact of Cd on the vascular tissue, a relatively high exposure was also used.

The level of supplementation with Zn was chosen to provide its important, but not too high intake. The mean daily intake of Zn in the animals supplemented with 30 mg Zn/L was 2.220 ± 0.084 mg/kg b.w. (mean ± SE) and 4.475 ± 0.407 mg/kg b.w. in the rats administered with 60 mg Zn/L [7]. The daily intake of Zn in particular groups treated with 30 and 60 mg Zn/L was elevated by 79% and 151%, respectively, compared to its consumption with the standard diet (1.253 ± 0.024 mg/rat (mean ± SE)). The measurements of Zn concentration in the serum (the main biomarker of the body status of Zn) (Supplementary Table S3), liver, brain, and bone tissue revealed that such elevation of the intake of this bioelement (both alone and under the treatment with Cd) did not result in its excessive content in the body [5,6,7].

At the end of the experiment, after overnight fasting, all rats were sectioned under intraperitoneal anesthesia with Vetbutal (pentobarbital sodium and pentobarbital 5:1, 30 mg/kg b.w.; Biowet, Pulawy, Poland). Various tissues and organs were dissected, including the abdominal aorta and whole blood (taken by cardiac puncture with or without anticoagulant—heparin; Biochemie, GmbH, Kundl, Austria). The whole blood taken without anticoagulant was centrifuged (MPW-350R centrifugator, Medical Instruments, Warsaw, Poland) after coagulation, and the serum was separated. The abdominal aorta and samples of the blood and serum were used in the present study. Once dissected, the aorta was rinsed thoroughly with an ice-cold (4 °C) physiological saline (0.9% sodium chloride) to remove the blood and gently drained using a filter paper. Slices of the aorta and samples of the blood subjected for the estimation of the expression of adhesive molecules were used immediately after collection. The biological material that was not used at once was stored frozen at −70 °C until the measurements.

The experimental model has been presented in detail in our previous reports [5,6,7,8,9,10].

### 2.5. Laboratory Procedures

All measurements using commercial kits were carried out according to the producers’ instructions. The precision of the measurements was expressed as the intra-assay coefficient of variation (CV).

The evaluation of oxidative/antioxidative status, inflammatory biomarkers, and the concentrations of TC, TG, and total protein was performed with the use of the MULTISCAN GO spectrophotometer (Thermo Scientific, Vantaa, Finland). The quantification of the expression of the adhesive molecules was carried out using Coulter Epics XL-MCL Cytometer (Beckman Coulter, Inc., Miami, FL, USA).

#### 2.5.1. Measurements in the Vascular Tissue 

##### Preparation of the Homogenates of the Vascular Tissue

The frozen slices (about 0.1–0.15 g) of the abdominal aorta, after thawing at room temperature, were weighted (with an accuracy of 0.0001 g) and homogenized in an ice-cold potassium phosphate buffer (50 mM, pH = 7.4; obtained by mixing 1 M potassium dihydrogen phosphate with 1 M dipotassium hydrogen phosphate (POCh) and distilled water) and butyl-hydroxytoluene (Sigma-Aldrich Gmbh, Steinheim, Germany) using a high-performance homogenizer (Ultra-Turrax T25, IKA, Staufen, Germany) to prepare 10% homogenates. After the centrifugation of the homogenates (MPW-350R centrifuge, Medical Instruments, Warsaw, Poland) at 4 °C (700× *g* for 20 min), the aliquots were rapidly separated and stored frozen at −70 °C until study.

##### Biochemical Measurements in the Homogenates of the Vascular Tissue

Biomarkers of the oxidative/antioxidative status (TAS and TOS), and inflammation (pro-inflammatory IL-1β and anti-inflammatory IL-10), as well as the concentrations of eNOS, TC, and TG, were determined in the supernatants of the homogenates of the vascular tissue. All the parameters, except for TC and TG, were adjusted for the concentration of total protein quantified (CV < 3%) using the BioMaxima Total Protein Kit (Lublin, Poland).

The ImAnOx (TAS) enzyme-linked immunosorbent assay (ELISA) kit by Immundiagnostik AG (Bensheim, Germany) was used to assay (CV < 4%) the TAS of the vascular tissue. The assay was based on the reaction of a defined amount of hydrogen peroxide (H_2_O_2_) with antioxidants present in the samples of the homogenates of the investigated vascular tissue. The residual H_2_O_2_ created products that absorbed the wavelength of 450 nm. The certified values of TAS in the control samples from the kit used for this parameter assay were 208–282 and 254–344 μmol/L, while the values measured by us reached 245.1 ± 13.1 and 261.1 ± 15.5 μmol/L (mean ± standard deviation (SD)), respectively. TOS was measured (CV < 4%) using the Immundiagnostik AG (Bensheim, Germany) PerOx (TOS) ELISA kit. The assay estimated the total amount of lipid peroxides present in the sample of the homogenates of the vascular tissue reacting with peroxidase at 450 nm. The values of TOS in the control samples reached 105–196 and 292–488 μmol/L, while the values quantified in our laboratory were 126.5 ± 3.5 and 403.6 ± 4.8 μmol/L (mean ± SD), respectively. The value of OSI was calculated based on the measurements of TOS and TAS as their ratio (OSI = TOS/TAS).

The concentrations of TC (CV < 3.5%) and TG (CV < 5%) were assayed using kits by BioMaxima (Lublin, Poland) and expressed in the calculation per g of the fresh tissue weight. eNOS was determined (CV < 7%) using the Rat Endothelial Nitric Oxide Synthase (eNOS) ELISA kit by MyBioSource (San Diego, CA, USA). The concentrations of IL-1β and IL-10 were measured (CV < 2% and 8%, respectively) using an IL-Iβ ELISA kit and IL-10 ELISA kit by R&D Systems (Minneapolis, MN, USA).

#### 2.5.2. Evaluation of Biomarkers of Inflammation in the Serum

The serum concentrations of IL-1β and IL-10 were determined (CV < 6% and 4%, respectively) using ELISA kits by R&D Systems (Minneapolis, MN, USA). The concentration of C-reactive protein (CRP) was measured (CV < 2%) with the use of Rat hsCRP ELISA kit by BioVendor (Modrice, Czech Republic), while VEGF was assayed (CV < 5%) with the use of the RayBio^®^ Rat VEGF-A ELISA Kit by RayBiotech, Inc. (Norcross, GA, USA).

#### 2.5.3. Estimation of the Expression of Adhesive Molecules on the Endothelial Cells of the Abdominal Aorta and Leukocytes in the Blood

To quantify the expression of adhesive molecules, such as platelet endothelial cell adhesion molecule-1 (PECAM-1), intercellular adhesion molecule-1 (ICAM-1), and L-selectin on the surface of the endothelial cells of the abdominal aorta, the tissue was prepared as follows. Small slices of the fresh aorta were chopped with a multi-blade tissue chopper. Next, the scraped tissue (vascular endothelium) was suspended in 2 mL of physiological buffered saline (PBS) in a tube with a conical bottom and mechanically treated by 20-time sucking back and forth with a plastic tip pipette (having a cut tip to provide an opening of 2–2.5 mm). Then, the endothelial cells were washed twice by centrifugation (MPW-350R centrifugator, Warsaw, Poland) at 200× *g* for 10 min. with 10 mL of PBS. At each step of the centrifugation, before filling up to 10 mL, cells were carefully resuspended in 3 mL of PBS by sucking the sediment several times back and forth with the plastic tip pipette. All the procedures were performed at 4 °C. After the last centrifugation, the further procedures were performed according to the appropriate instructions provided with the commercial diagnostic kits.

A total of 100 μL of the epithelial cell suspension or whole blood (fresh blood taken by cardiac puncture on heparin was used to evaluate the leukocytic expression of the adhesive molecules) were incubated with appropriate monoclonal antibodies (anti-CD31, anti-CD54, or anti-CD62) in the amount of 5 μL per sample for 15 min at room temperature. Then, in the case of the samples of the whole blood, erythrocytes were lysed with the use of ImmunoPrep Lysing Solution (Beckman Coulter, Lakeview, IN, USA). The expression of the adhesive molecules was quantified by flow cytometry method using Fluorescein Isothiocyanate (FIT)-Conjugated Mouse Anti-Rat CD-54 (ICAM-1) Monoclonal Antibody, PR-Phycoerythrin (R-PE)-Conjugated Mouse Anti-Rat CD45 (Leukocyte Common Antigen) Monoclonal Antibody, R-Phycoerythrin (R-PE)-Conjugated Mouse Anti-Rat CD31 (PECAM-1) Monoclonal Antibody, and Fluorescein Isothiocyanate (FIT)-Conjugated Hamster Anti-Rat CD62L (L-selectin, LECAM-1) Monoclonal Antibody (BD Biosciences Pharmingen, San Diego, CA, USA). The expression of ICAM-1 and L-selectin was evaluated on the endothelial cells and leukocytes, while the expression of PECAM-1 was detectable only on the endothelial cells. The results are presented as the percentage of cells expressing the adhesive molecules. The cells were counted under a light microscope (Olympus, Tokyo, Japan).

### 2.6. Statistical Analysis

All statistical calculations were performed using the Statistica 13 package (StatSoft, Tulsa, OK, USA). Shapiro-Wilk test showed no normal distribution of the data; therefore, the results are presented in tables as a median and minimum and maximum for 8 rats in each group (the results are also presented in figures showing the directions and extent of differences between experimental groups provided as Supplementary Material). To recognize any statistically significant differences among the experimental groups, a nonparametric signed-rank Kruskal–Wallis test was performed (*p* < 0.05). A Kruskal–Wallis post hoc test was completed to check the existence of a statistically significant difference (*p* < 0.05) between every two medians. Statistically significant differences in the measured parameters concerning the control group, Zn30 group, Zn60 group, Cd5 group, Cd5 + Zn30 group, Cd5 + Zn60 group, and Cd50 group are presented. A linear regression analysis was conducted to show the relationships between the investigated parameters, as well as between them and the concentrations of Cd in the blood and Zn in the serum of these animals, assayed in our previous study [7]. The results of the analysis of regression are presented as a β coefficient (the degree of change in the dependent variable for every 1-unit of change in the independent variable), *R*^2^ (shows what percentage of one variable explains the variability of the other variable; it takes values from 0 to 1), and the level of statistical significance (*p*). Statistically significant dependence between two variables was discerned at the value of the β coefficient for which *p* < 0.05.

## 3. Results

### 3.1. The Impact of Zn and/or Cd on the Oxidative/Antioxidative Status of the Vascular Tissue of the Abdominal Aorta

In the groups of rats maintained for 6 months on the drinking water containing 30 or 60 mg Zn/L, TAS of the vascular tissue reached the same values as in the control group, while TOS and OSI were lower (by 60–67%) (Table 2). The exposure to 5 and 50 mg Cd/L led to a decrease (by 40% and 45%, respectively) in the TAS (Table 2). The value of this parameter in all groups co-administered with Cd and Zn was at the proper level except for the Cd5 + Zn60 group in which it was decreased by 48% (Table 2).

In the Cd5 group, the TOS of the vascular tissue was unaffected; however, in the Cd5 + Zn60 group, it was lower compared to the control group (by 71%) and the Cd5 group (by 77%). In the group exposed to 50 mg Cd/L, the vascular tissue TOS was three times elevated (Table 2). In the animals supplemented with Zn under the higher intoxication with Cd, TOS was even lower (by 59% in the Cd50 + Zn30 and 61% in the Cd50 + Zn60 group) than in the control group and lower (by 86% and 87%, respectively) compared to the Cd50 group (Table 2).

The treatment with 5 mg Cd/L led to a 2-fold increase in the value of OSI, while the exposure to 50 mg Cd/L made it six times higher than that in the control group. However, as in the case of TAS and TOS, there was no difference in the value of OSI between the Cd5 and Cd50 groups (Table 2). In the rats supplemented with 30 and 60 mg Zn/L under the treatment with Cd, OSI did not differ from the control group and was lower (by 74–91%) compared to the respective group that did not receive this bioelement under the treatment with Cd (Table 2).

### 3.2. The Impact of Zn and/or Cd on the Concentrations of TC, TG, and eNOS in the Vascular Tissue of the Abdominal Aorta

The administration of Zn alone did not affect the concentrations of TC, TG, and eNOS in the vascular tissue (Table 3). There were no differences in the concentrations of these parameters between the Cd5 and Cd50 groups (Table 3).

The exposure to 5 and 50 mg Cd/L increased the concentration of TC (by 48% and 61%, respectively) compared to the control, while in the animals co-administered with 30 and 60 mg Zn/L, the value of this parameter did not differ compared to the control group and in the Cd50 + Zn30 and Cd50 + Zn60 groups it was lower (by 36% and 29%, respectively) than in the Cd50 group (Table 3).

The intoxication with 5 and 50 mg Cd/L led to an elevation in the concentration of TG (by 61% and 45%, respectively) (Table 3). In the animals co-administered with Zn, the concentration did not differ compared to the control group. Moreover, in the Cd50 + Zn30 group and Cd50 + Zn60 group, it was lower (by 36% and 31%, respectively) compared to the Cd50 group (Table 3).

The concentration of eNOS was unchanged in the groups treated with 5 mg Cd/L alone and together with 30 or 60 mg Zn/L (Table 3). The higher exposure to this xenobiotic led to a 2.5-fold growth in the concentration of this parameter, whereas in the animals co-administered with Zn (30 and 60 mg/L) the concentration was in the range of the proper values and was lower (by 71% and 72%, respectively) than in the Cd50 group (Table 3).

### 3.3. The Impact of Zn and/or Cd on the Concentrations of Biomarkers of Inflammation in the Vascular Tissue of the Abdominal Aorta and Serum

The administration of Zn alone had no impact on the concentrations of IL-1β and IL-10 in the vascular tissue of the abdominal aorta and serum (Table 4), as well as on the concentration of CRP in the serum (Table 4). There was no difference in the values of the inflammatory biomarkers in the vascular tissue and serum between the Cd5 and Cd50 groups (Table 4).

The vascular tissue concentration of IL-1β in the animals exposed to 5 and 50 mg Cd/L was elevated (23- and 60-fold, respectively) compared to the control (Table 4). The concentration of this interleukin in the Cd5 + Zn30 and Cd5 + Zn60 groups was also increased (37- and 31-fold, respectively), whereas in the case of this element co-administration during the higher exposure to Cd did not differ compared to the control and was lower (by 71%) than in the Cd50 group (Table 4).

The intoxication with 5 and 50 mg Cd/L did not affect the concentration of IL-10 in the vascular tissue (Table 4). The concentration of this interleukin in the Cd5 + Zn30 group was increased compared to the control group (2.2 times), whereas in the Cd50 + Zn30 and Cd50 + Zn60 groups it was higher (from 2.2- to 2.6-fold) compared to the control and Cd50 groups (Table 4).

The administration of the drinking water containing 5 mg Cd/L alone or together with Zn (30 and 60 mg/L) led to an increase (from 6- to 9-fold) in the concentration of IL-1β in the serum (Table 4). In the animals intoxicated with 50 mg Cd/L, the serum concentration of this pro-inflammatory cytokine was also elevated (17 times), while in the case of simultaneous supplementation with Zn, the concentration was within the range of proper values and was lower (by 76%) than in the Cd50 group (Table 4).

The serum concentration of IL-10 was similarly decreased at both levels of Cd intoxication (by 73% and 74%), whereas in the case of co-administration with 30 or 60 mg Zn/L it was within the range of values assayed in the control animals (Table 4). The serum concentration of IL-10 in the Cd5 + Zn30 group was 2-fold higher than in the Cd5 group (Table 4).

The concentration of CRP in the serum after the 6-month treatment with 5 mg Cd/L was higher (4-fold) compared to the control group, whereas under the co-administration of Zn it did not differ from both the control and Cd5 groups (Table 4). The exposure to 50 mg Cd/L increased (7-fold) the concentration of this protein (Table 4). In the animals co-supplemented with 30 mg Zn/L, the concentration did not differ compared to the control group and was lower (by 64%) than in the Cd50 group; however, in the Cd50 + Zn60 group, it was higher (3.5-fold) than in the control group (Table 4).

### 3.4. The Impact of Zn and/or Cd on the Concentration of VEGF in the Serum

The supplementation with Zn alone did not influence the concentration of VEGF in the serum (Table 5). The exposure to Cd did not affect the concentration of VEGF irrespective of the level of intoxication (Table 5). In the group co-administered with 5 mg Cd/L and 60 mg Zn/L, the concentration of VEGF was lower by 77% compared to the respective group treated with Cd alone (Table 5). In the animals supplemented with 30 and 60 mg Zn/L under the higher exposure to Cd, the concentration of VEGF was lower (by 70% and 90%, respectively) in comparison to the Cd50 group; however, it was within the range of the values determined in the control animals (Table 5).

### 3.5. The Impact of Zn and/or Cd on the Expression of Adhesive Molecules on the Endothelial Cells of the Abdominal Aorta and Leukocytes in the Blood

The administration of 30 or 60 mg Zn/L alone did not affect the expression of ICAM-1, PECAM-1, and L-selectin on the endothelial cells (Table 6). The expression of all investigated adhesive molecules on these cells was lower in the groups exposed to 5 and 50 mg Cd/L (by 77–94%), as well as in the animals co-administered with Cd and Zn (by 89–98%) as compared to the control group (Table 6). The expressions of PCAM-1 in the Cd50 + Zn30 and Cd50 + Zn60 groups and L-selectin in the Cd50 + Zn60 group were even lower (by 62–84%) than in the Cd50 group (Table 6).

The administration of Zn and Cd alone did not affect the expression of ICAM-1 and L-selectin on leukocytes (Table 7). The leukocyte expression of ICAM-1 was elevated (13- to 20-fold) in the Cd5 + Zn30, Cd5 + Zn60, and Cd50 + Zn30 groups, while in the Cd50 + Zn60 group it did not differ compared to the control group (Table 7). The co-administration of Cd and Zn decreased the expression of L-selectin compared to the control group (by 75–93%) and respective Cd group (by 68–91%) (Table 7).

### 3.6. Mutual Relationships between the Investigated Parameters, as Well as between These Parameters and the Concentrations of Cd in the Blood and Zn in the Serum

Numerous mutual dependencies were noted between the parameters estimated in the vascular tissue of the abdominal aorta (Table 8). TAS and TOS correlated negatively or positively, respectively, with OSI; however, there was no dependence between TAS and TOS (Table 8). The vascular tissue TAS negatively correlated with the concentrations of TC, TG, and IL-1β and positively with the endothelial expression of PECAM-1, ICAM-1, and L-selectin. There were strong positive correlations between TOS and OSI and all parameters evaluated in the abdominal aorta except for a negative correlation between these indices of the oxidative status and the concentration of IL-10 and a lack of dependence between them and the expression of adhesive molecules (Table 8). The vascular concentrations of TC, TG, IL-1β, and IL-10 negatively correlated with the expression of adhesive molecules, except for a lack of dependence between TG and L-selectin (Table 8). The concentrations of TC and TG positively correlated with the concentrations of eNOS and IL-1β, while between the concentrations of TG and IL-10 a negative dependence was noted. The concentration of eNOS positively correlated with that of IL-1β and negatively with IL-10. Positive dependence was noted between the concentrations of TC and TG. Moreover, mutual positive dependencies occurred between the endothelial expression of PECAM-1, ICAM-1, and L-selectin (Table 8).

Numerous relationships were noted between the serum concentrations of CRP, IL-1β, IL-10, and VEGF and parameters determined in the vascular tissue (Table 9). TAS negatively correlated with the serum concentrations of CRP, IL-1β, and VEGF, while between the aorta TOS, OSI, TC, TG, and eNOS and these variables in the serum positive dependencies occurred (Table 9). The serum concentration of IL-10 positively correlated with TAS and negatively with TOS, OSI, TC, TG, eNOS, IL-1β, and IL-10 and positively with the expression of adhesive molecules (Table 9). A negative relationship was revealed between the endothelial expression of adhesive molecules and the serum concentrations of CRP and IL-1β (Table 9).

Numerous positive and negative relationships were noted between the investigated parameters and the concentration of Cd in the blood and Zn in the serum (Table 10). The values of all measured variables, except for the concentration of TG in the vascular tissue, expression of ICAM-1 on leukocytes, and the concentration of VEGF in the serum, correlated negatively (vascular tissue TAS, expression of adhesive molecules on the endothelial cells, expression of L-selectin on leukocytes, and the serum concentration of IL-10) or positively (TOS and OSI, the concentrations of TC, eNOS, IL-1β, and IL-10 in the vascular tissue, and the serum concentrations of IL-1β and CRP). Positive dependencies were revealed between the serum concentration of Zn and the vascular tissue TAS, the endothelial expression of all adhesive molecules, and the serum concentration of IL-10 (Table 10). The vascular tissue TOS and OSI, the concentrations of TC, TG, and IL-1β, as well as the serum concentrations of IL-1β and CRP negatively correlated with the serum concentration of Zn (Table 10).

## 4. Discussion

The most important finding of the present study is the protective impact of the supplementation with Zn on the vascular tissue in a rat model of human environmental exposure to Cd in industrialized countries and a proposal of the possible mechanism of this protection. Moreover, the study provides unquestionable evidence that exposure to Cd, resulting in its concentration in the blood comparable to that nowadays noted in inhabitants of industrialized countries, has an unfavorable impact on the vascular tissue.

The measurements of the markers of oxidative-reductive status and inflammation showed that moderate and relatively high intoxication with this heavy metal led to the development of oxidative stress and stimulated inflammatory processes in the vascular tissue. Oxidative stress and inflammation are considered to be the potential common etiological factors of cardiovascular disorders [20,23,24,26,27,29,41,42]. Cd may induce the excessive formation of reactive oxygen species (ROS) and free radicals (FR) by, e.g., decreasing the activity of antioxidative enzymes (including Zn-dependent superoxide dismutase—SOD) and the concentration of non-enzymatic antioxidants (including reduced glutathione—GSH) and induction of the activity of oxidases, and dysfunction of mitochondria [8,9,23,28,43]. Moreover, this xenobiotic may promote oxidative stress in the vascular endothelium by enhancing the generation of angiotensin II which can stimulate nicotinamide adenine dinucleotide phosphate (*NADPH*) *oxidase* [27]. Increased formation of ROS and RF may cause damage to the cellular macromolecules, especially lipids, leading to necrosis and apoptosis of the endothelial cells and eventually resulting in vascular dysfunction [25,26,27,28,41]. Our finding that Cd increases the concentrations of CRP and IL-1β and decreases that of IL-10 follows the outcomes of other authors [17,44,45]. The fact that Cd at both levels of exposure decreased the concentration of IL-10 in the serum but did not affect its concentration in the vascular tissue together with the available literature data [45,46] may show that the impact of this metal on the concentration of this cytokine is dose-, time-, and tissue-dependent. Detailed analysis of the results of the present study, including the relationships between the values of all evaluated markers of the oxidative-reductive status and inflammation and Cd concentration in the blood, allows for the conclusion that oxidative stress and vascular inflammation that are recognized as major triggers for the cardiovascular diseases may also be the main triggers for the impact of Cd on the blood vessels and circulatory system.

An important risk factor for cardiovascular diseases is also excessive lipid accumulation in the walls of the blood vessels [17,47]. The finding that the exposure to Cd led to an increase in the concentrations of TC and TG in the vascular tissue together with the dependencies between these parameters and biomarkers of inflammation indicate that this toxic metal may promote vascular inflammation and atherosclerosis and in this way lead in consequence, or at least contribute, to the development of cardiovascular disorders. Studies by other authors, conducted in animal models, also showed that intoxication with Cd may result in alterations in the lipid profile [10,26,48].

Cd may affect the cardiovascular system also via influencing the activity of eNOS and thus the vascular production of NO, which plays an important role in maintaining cardiovascular homeostasis [28,30]. The elevation in the concentration of eNOS in the vascular tissue noted at the higher level of Cd intoxication confirms this mechanism and it may stem from the Cd-mediated growth in TOS and consequently the development of oxidative stress [49], reflected in the enhanced value of OSI. Al-Naemi and Das [50] showed that chronic exposure to this element induced the expression of eNOS in the thoracic aorta; however, other authors have reported a decrease in this enzyme expression in the endothelial cells [30] and thoracic aorta [28].

A key stage in the development of atherosclerotic lesions is the increase in the adhesion of leukocytes (especially monocytes) to the endothelial surface, which is related to the presence of adhesive molecules. These molecules are proteins that appear on the endothelial cells and interact with antigens present on leukocytes that allow them to adhere to the endothelium [41,42]. Adhesive molecules appear during the activation of endothelial cells by pro-inflammatory cytokines (e.g., IL-1β, IL-6, and tumour necrosis factor α—TNF-α) [41,42]. Thus, taking into account the findings of the present research and some results of our previous studies in these animals [6,10], an unexpected outcome is the finding that Cd decreased the expression of ICAM-1, PECAM-1, and L-selectin on the endothelial cells. This is also contrary to the results of other authors who reported an increase in the expression of ICAM-1 due to the treatment with Cd in the thoracic aorta [28] and liver [51] of experimental animals and in an in vitro study [29]. Interestingly, experimental studies on human aortic endothelial cells do not confirm the pro-inflammatory effect of Cd mediated by activation of ICAM-1 or pro-inflammatory genes [52]. Sundaresan et al. [53] demonstrated the downregulation of the expression of PECAM-1 in the heart tissue of rats exposed to Cd (15 mg CdCl_2_/kg b.w. in drinking water for 60 days). Shi et al. [54] reported that enhanced expression of PECAM-1 improved the function of cardiac microvascular endothelial cells in angiotensin II-induced pathological cardiac hypertrophy. Therefore, it can be assumed that the Cd-induced decrease in the endothelial expression of this adhesive molecule may also be an important factor contributing to the injury of the blood vessels by this xenobiotic. In the available literature, there are no data on the impact of Cd on the vascular expression of L-selectin; however, Mousa [51] revealed induction of E-selectin by Cd in the liver of rats. Because adhesive molecules are proteins, it seems possible that the Cd-caused decrease in their expression might be an effect of its unfavourable impact on the metabolism of proteins, including their biosynthesis [12].

It is also worth emphasizing that, although, there were no differences in the values of all assayed parameters between the Cd5 and Cd50 groups, the relationships between these parameters and Cd concentration in the blood confirm that the destroying impact will intensify with the increasing body burden of this xenobiotic. As it is evident in the tables presenting the results concerning the vascular tissue TOS, OSI, and the concentrations of eNOS and IL-1β, as well as the serum concentrations of IL-1β and CRP, the medians of these parameters in the Cd50 group reached higher values than in the Cd5 group. The lack of statistically significant differences in these parameters might result from the relatively wide ranges of their values in particular groups.

The most valid outcome of the present investigation is that the enhanced intake of Zn prevented most of the Cd-induced alterations in the vascular tissue or at least weakened them and improved some parameters unaffected by Cd, as well as exerted an anti-inflammatory effect in the serum. The revelation that the co-administration of this bioelement under exposure to Cd prevented the development of oxidative stress, retention of TC and TG, and the increase in the concentration of eNOS in the vascular tissue, as well as protected (partially or totally) against some alterations of the inflammatory biomarkers in the vascular tissue and serum provides strong evidence for its protective impact against vascular toxicity of Cd. It is difficult to explain why the supplementation with 60 mg Zn/L did not protect against the 5 mg Cd/L-induced decrease in the vascular tissue TAS, while the effect was observed as a result of the co-administration of 30 mg Zn/L, and this element, at both levels of supplementation, prevented the 5 mg Cd/L-caused decrease in this bioelement concentration in the serum [7]. Taking into account that oxidative stress and inflammation, as well as the disturbances in lipid metabolism and eNOS activity, are among the leading causes of vascular damage [20,23,24,26,27,41,42], it can be suspected that the administration of Zn could also protect from structural and functional changes in the vascular tissue; however, this issue requires further investigation. Taking into account the relationship between inflammatory processes and adhesive molecules expression [41] and the noted beneficial impact of Zn in the rats exposed to Cd, it is difficult to explain why the administration of 30 and 60 mg Zn/L did not prevent the influence of this xenobiotic on the vascular expression of adhesive molecules and that the expressions of ICAM-1 and L-selectin on leukocytes, which were unchanged by the exposure to Cd or supplementation with Zn alone were modified in the case of co-administration of both elements.

The protective effect of Zn against the harmful impact of Cd on the vascular tissue may primarily be an outcome of its antioxidative and anti-inflammatory properties [1,5,6,8,9,25,36,39]. Zn is a co-factor for SOD [1,39] and may influence the expression of transcription factors and genes encoding antioxidants, induce the synthesis of metallothionein, modulate the nuclear factor erythroid 2-related factor (Nrf2) signaling, and mitigate the toxic action of transition metals possessing pro-oxidative properties, such as iron (II) and copper (I) [1,36,39]. The anti-inflammatory properties of this element are mainly due it its direct impact on the immune cells, transcription factors, such as nuclear factor kappa-light-chain-enhancer of activated B cells (NF-κB), and expression of genes for pro-inflammatory molecules [1,38]. Previously we have revealed that the supplementation with Zn (30 and 60 mg/L) protected (entirely or partially) against Cd-induced (5 and 50 mg/L) increase in the serum concentration of TNF-α [6], prevented an elevation of the concentrations of TC, free fatty acids, lipid peroxides, and low-density lipoprotein cholesterol (LDL-C) and a decrease in the concentrations of phospholipids and high-density lipoprotein cholesterol (HDL-C) in the serum, as well as mitigated a Cd-induced increase in the serum concentration of F2-isoprostane [10]. The outcomes of the present study together with the previous findings [6,10] prove the antiatherogenic action of Zn under exposure to Cd.

The findings of the present study show that the beneficial effect of Zn administration on the cardiovascular system under exposure to Cd may also consist of decreasing the serum concentration of VEGF, being a key factor in normal and pathological angiogenesis. Because VEGF represents an important pro-angiogenic activity, having a mitogenic and an anti-apoptotic effect on the endothelial cells, increasing the vascular permeability, promoting cell migration, etc. [55], the decrease in its concentration due to the enhanced Zn intake under exposure to Cd is a beneficial effect. Positive relationships between the serum concentration of VEGF and the vascular tissue TOS and OSI, the concentrations of TC, TG, eNOS, and IL-1β, as well as the negative dependence between the concentration of this growth factor and the vascular tissue TAS, confirm that the protective effect of Zn on the vessels may be, although to a minor extent, related to the impact on the concentration of VEGF in the serum.

Although we did not investigate the influence of Zn on Cd accumulation in the vascular tissue, it can be suspected that the beneficial effect of this necessary element is the result of competition between these metals [36]. It is known that ions of Cd (Cd^2+^) may use transporter systems for ions of Zn (Zn^2+^), including ZP8, ZIP14, and divalent metal transporter 1 (DMT1) to enter the cells and that Zn^2+^ inhibits the cellular uptake of Cd^2+^ [36,56]. Moreover, because in the animals supplemented with 30 and 60 mg Zn/L during the exposure to 50 mg Cd/L, the blood concentration of Cd was lower (by 17% and 14%, respectively) than in the ones not receiving this bioelement [7], it seems possible that the beneficial impact of Zn might also be related to its interaction with Cd resulting in a lower concentration of the latter element in the blood. Because the blood is in direct contact with the epithelium of the blood vessels, a lower concentration of Cd in this biological fluid means that a lesser amount of this xenobiotic is available to exert the toxic impact on the vascular epithelium. Numerous relationships noted between the blood concentration of Cd and the parameters measured in the vascular tissue of the abdominal aorta confirm the dependence between the blood concentration of this toxic element and its damaging influence on the vessels and at the same time the possibility of the above mechanism of the protective impact of Zn. Moreover, the fact that the supplementation with 30 and 60 mg Zn/L under the exposure to 5 mg Cd/L had no impact on the blood concentration of this xenobiotic confirms that the protective impact of Zn was related to its direct action.

It is also worth highlighting that a valid achievement of the present research is showing that the enhancement in the administration of Zn by 79% and 151% did not induce disturbances of the investigated parameters in the vascular tissue. Moreover, the findings indicate that both levels of Zn dosage were effective in combating the toxic impact of Cd on the vascular tissue. However, taking into account the results of our previous research showing that supplementation with 30 mg Zn/L was more effective in mitigating Cd toxicity in the liver than 60 mg Zn/L [6], it may be suspected that a 79% increase in its administration is enough to protect target organs and systems from the toxic action of this xenobiotic and at the same time does not pose a risk of negative outcomes of excessive intake of Zn. The percentage enhancement of Zn intake, revealed by us to protect from the toxic impact of Cd on the cardiovascular system and numerous other effects of its action [5,6,7,8,9,10] in male rats, seems not to be high taking into account that the established Tolerable Upper Intake Level for this bioelement is 40 mg/day for men and women and reaches 250–364% and 400–571% of the Recommended Dietary Allowance (11–16 mg/day for men and 7–10 mg/day for women), respectively [57,58].

We are aware of not only the achievements but also of the limitations of our study. The experiment was conducted on male rats, therefore its findings refer, first of all, to males. Nevertheless, it is reasonable to assume that Cd may have at least a similar effect on vascular tissue in females. Although taking into account that females generally have higher Cd concentration in the blood and are more susceptible to the toxicity of this xenobiotic than males [12], the changes induced by Cd probably could be more severe, which could also affect the efficacy of the protection provided by Zn. Thus, it seems necessary to investigate the impact of Zn under moderate exposure to Cd in females. Another limitation of the study is the impossibility of a proper explanation of all mechanisms of the impact of both elements on the vascular tissue, especially the impact of Cd on the expression of adhesive molecules on the vascular epithelium and why there was no difference in the influence of this xenobiotic at the moderate and relatively high exposure.

In summary, the findings of the present research show, for the first time, in the rat model of moderate and relatively high environmental human exposure to Cd, that this xenobiotic is a potent factor contributing to the damage to the blood vessels and that an enhancement of Zn intake by 79% and 151% protects against Cd-mediated development of oxidative stress, inflammatory processes, and its proatherogenic action in the vascular tissue at the abdominal aorta. It is worth underlining that the increase in the intake of Zn by 79% may be sufficient to provide this protection. An important result of this investigation is also revealing that enhanced intake of this essential micronutrient does not pose a danger of disturbances in the vascular tissue. Since damage to the endothelial function is a key element in the development of atherogenic lesions, the results indicate a possible atherogenic effect of Cd at its blood concentrations nowadays recorded in the general population. The finding that Zn protects from changes in the endothelium confirms the anti-atherogenic effect of this essential element and its ability to protect against the atherogenic effect of Cd. Considering the revealed efficacy of Zn in preventing the injurious action of Cd on the vascular tissue and our previous results in the same animals [5,6,7,8,9,10], this bioelement should be recognized as a possible factor to combat the toxicity of this heavy metal at oral exposure, including its impact on the cardiovascular system, especially blood vessels. Thus, our findings have not only scientific value but also important practical implications.

## Figures and Tables

**Table 1:** The daily intakes of Cd and Zn in particular experimental groups [7] ^1^.

Experimental Group	Cd Intake (mg/kg b.w./24 h)	Zn Intake (mg/kg b.w./24 h)
Control	0	0
Zn30	0	1.26–3.67
Zn60	0	2.40–6.41
Cd5	0.222–0.731	0
Cd5 + Zn30	0.243–0.745	1.33–3.57
Cd5 + Zn60	0.260–0.740	2.41–6.67
Cd50	1.850–4.340	0
Cd50 + Zn30	2.000–4.370	1.41–3.98
Cd50 + Zn60	2.000–4.440	2.70–7.14

^1^ Data are presented as the minimum and maximum intake of Cd and Zn in particular experimental groups during their 6-month administration. b.w., body weight.

**Table 2:** TAS, TOS, and OSI in the vascular tissue of the abdominal aorta in particular experimental groups ^2^.

Experimental Group	TAS (nmol/mg Protein)	TOS (nmol/mg Protein)	OSI
Control	87.5 78.1–108.0	11.78 9.82–13.60	0.126 0.122–0.142
Zn30	102.2 98.5–111.2	4.63 ^a^* 2.70–6.36	0.041 ^a^^†^ 0.019–0.089
Zn60	85.4 74.1–108.6	4.31 ^a^* 3.18–5.34	0.051 ^a^^†^ 0.031–0.058
Cd5	52.8 ^a^* ^b^^‡ c^* 30.5–60.5	14.98 ^b^* ^c^* 11.31–17.05	0.328 ^a^^† b^^‡ c‡^ 0.294–0.373
Cd5 + Zn30	80.1 60.4–98.4	5.32 4.35–8.50	0.074 ^d^* 0.056–0.091
Cd5 + Zn60	45.3 ^a^^† b^^‡ c†^ 30.4–50.2	3.46 ^a^^† d†^ 3.04–5.13	0.079 ^d^* 0.069–0.159
Cd50	48.1 ^a^^† b^^‡^ 38.6–67.1	33.43 ^a^^† b‡ c‡ e^* ^f^^‡^ 7.38–46.14	0.669 ^a^^‡ b‡ c‡ e† f^* 0.598–0.775
Cd50 + Zn30	56.0 ^b^^†^ 45.1–98.7	4.83 ^a^* ^d^* ^g^^†^ 0.93–5.78	0.067 ^d^* ^g^^†^ 0.0570–0.102
Cd50 + Zn60	54.6 ^b^^‡^ 36.4–64.3	4.57 ^a^* ^g^^†^ 1.54–6.84	0.095 ^g^* 0.074–0.110

^2^ Data represent the median value and minimum and maximum for 8 rats in each experimental group. Statistically significant differences (nonparametric Kruskal–Wallis test): ^a^ vs. Control group; ^b^ vs. Zn30 group; ^c^ vs. Zn60 group; ^d^ vs. Cd5 group; ^e^ vs. Cd5 + Zn30 group; ^f^ vs. Cd5 + Zn60 group; ^g^ vs. Cd50 group; * *p* < 0.05; ^†^ *p* < 0.01; ^‡^ *p* < 0.001. The directions and extent of differences between the experimental groups are presented in Supplementary Figure S1.

**Table 3:** The concentrations of TC, TG, and eNOS in the vascular tissue of the abdominal aorta in particular experimental groups ^2^.

Experimental Group	TC (mg/g Tissue)	TG (mg/g Tissue)	eNOS (ng/mg Protein)
Control	1.048 1.012–1.134	1.919 1.519–2.047	9.809 6.482–15.94
Zn30	1.054 1.016–1.215	1.906 1.792–2.059	9.840 8.210–12.01
Zn60	1.088 0.969–1.165	1.779 1.558–2.067	9.579 7.463–12.84
Cd5	1.550 ^a^^‡ b^^† c^^†^ 1.496–1.731	3.092 ^a^^† b^* ^c^^‡^ 2.363–3.882	11.50 7.147–16.55
Cd5 + Zn30	1.296 1.221–1.579	2.064 1.941–2.217	9.267 5.199–13.85
Cd5 + Zn60	1.1914 1.037–1.215	2.243 1.858–2.743	10.17 4.073–14.99
Cd50	1.689 ^a^^‡ b^^† c^^‡^ 1.507–1.721	2.784 ^a^^† b^* ^c^^†^ 2.370–3.143	24.43 ^a^* ^e^* ^f^* 16.50–38.53
Cd50 + Zn30	1.076 ^d^^† g†^ 0.908–1.338	1.770 ^d^^‡ g^^‡^ 1.626–1.994	7.029 ^g^^‡^ 3.393–10.10
Cd50 + Zn60	1.198 ^g^* 0.808–1.353	1.932 ^d^^† g^^†^ 1.546–2.042	6.851 ^g^^‡^ 5.588–14.23

^2^ Data represent the median value and the minimum and maximum for 8 rats in each experimental group. Statistically significant differences (nonparametric Kruskal–Wallis test): ^a^ vs. Control group; ^b^ vs. Zn30 group; ^c^ vs. Zn60 group; ^d^ vs. Cd5 group; ^e^ vs. Cd5 + Zn30 group; ^f^ vs. Cd5 + Zn60 group; ^g^ vs. Cd50 group; * *p* < 0.05; ^†^ *p* < 0.01; ^‡^ *p* < 0.001. The directions and extent of differences between the experimental groups are presented in Supplementary Figures S2 and S3.

**Table 4:** The concentrations of biomarkers of inflammation in the vascular tissue of the abdominal aorta and serum in particular experimental groups ^2^.

Experimental Group	Vascular Tissue		Serum		
	IL-1β (pg/mg Protein)	IL-10 (pg/mg Protein)	IL-1β (pg/mL)	IL-10 (pg/mL)	CRP (μg/mL)
Control	12.69 9.04–16.51	22.05 18.72–22.87	25.37 18.08–33.02	109.7 93.2–137.2	67.55 53.81–90.84
Zn30	12.30 8.77–16.38	27.24 20.83–29.44	24.60 17.54–33.13	136.2 104.1–147.2	56.29 41.89–64.18
Zn60	12.90 10.48–15.72	24.70 22.88–26.31	25.80 20.97–31.43	123.5 114.4–131.5	86.21 44.13–116.8
Cd5	288.1 ^a^* ^b^* ^c^* 237.0–422.1	26.11 22.06–41.97	144.0 ^a^* ^b^* ^c^* 118.5–211.0	31.33 ^a^^† b^^‡ c^^‡^ 26.47–50.37	275.0 ^a^^‡ b^^‡ c^^†^ 236.9–349.9
Cd5 + Zn30	466.9 ^a^^† b^^† c^^†^ 306.9–578.9	48.63 ^a^^‡^ 34.89–57.27	233.4 ^a^^† b^^† c^^†^ 153.5–289.5	60.79 ^d^* 43.61–71.59	108.8 95.95–171.1
Cd5 + Zn60	395.4 ^a^^† b^^† c^^†^ 314.9–664.7	34.97 26.57–50.56	197.7 ^a^^† b^^† c^^†^ 157.5–332.3	43.72 ^b^* 33.21–63.20	154.0 133.1–215.3
Cd50	761.4 ^a^^‡ b^^‡ c^^‡^ 568.4–898.3	20.34 14.54–24.60	423.0 ^a^^‡ b^^‡ c^^‡^ 315.8–499.1	28.18 ^a^^‡ b^^‡ c^^‡^ 17.45–32.88	484.8 ^a^^‡ b^^‡ c^^‡ e†^ 430.5–605.3
Cd50 + Zn30	221.1 ^g^* 174. 7–313.3	47.30 ^a^^‡ e^^‡ g^^‡^ 36.23–62.08	100.5 ^g^* 79.4–142.4	39.42 ^b^^† c^* 30.19–51.73	179.5 ^b^* ^g^^†^ 146.2–190.5
Cd50 + Zn60	222.8 ^g^* 202.4–461.3	53.34 ^a^^‡ b^* ^c^* ^g^^‡^ 39.45–88.57	101.3 ^g^* 92.0–209.7	44.45 ^b^* 32.88–73.81	237.0 ^a^^† b‡ c^* 205.4–217.4

^2^ Data represent the median value and the minimum and maximum for 8 rats in each experimental group. Statistically significant differences (nonparametric Kruskal–Wallis test): ^a^ vs. Control group; ^b^ vs. Zn30 group; ^c^ vs. Zn60 group; ^d^ vs. Cd5 group; ^e^ vs. Cd5 + Zn30 group; ^g^ vs. Cd50 group; * *p* < 0.05; ^†^ *p* < 0.01; ^‡^ *p* < 0.001. The directions and extent of differences between the experimental groups are presented in Supplementary Figures S4 and S5.

**Table 5:** The concentration of VEGF in the serum in particular experimental groups ^2^.

Experimental Group	VEGF (pg/mL)	
	Median	Minimum and Maximum
Control	0.612	0.294–1.229
Zn30	0.258	0.055–0.453
Zn60	0.385	0.067–0.730
Cd5	1.744 ^b^* ^c^*	0.723–9.146
Cd5 + Zn30	0.562	0.083–0.928
Cd5 + Zn60	0.400 ^d^*	0.095–0.955
Cd50	1.398 ^b^* ^c^* ^f^*	1.131–3.606
Cd50 + Zn30	0.409 ^g^*	0.153–0.760
Cd50 + Zn60	0.140 ^d^^† g†^	0.063–0.951

^2^ Data represent the median value and the minimum and maximum for 8 rats in each experimental group. Statistically significant differences (nonparametric Kruskal–Wallis test): ^b^ vs. Zn30 group; ^c^ vs. Zn60 group; ^d^ vs. Cd5 group; ^f^ vs. Cd5 + Zn60 group; ^g^ vs. Cd50 group; * *p* < 0.05; ^†^ *p* < 0.01. The directions and extent of differences between the experimental groups are presented in Supplementary Figure S6.

**Table 6:** The expression of PECAM-1, ICAM-1, and L-selectin on the endothelial cells of the abdominal aorta in particular experimental groups ^2^.

Experimental Group	PECAM-1 (%)	ICAM-1 (%)	L-Selectin (%)
Control	4.65 3.40–5.10	2.85 1.20–3.20	3.41 2.50–4.33
Zn30	1.60 0.90–2.90	0.81 0.32–1.50	0.75 0.56–1.22
Zn60	1.78 0.90–3.00	0.68 0.33–2.49	1.48 0.80–2.79
Cd5	0.55 ^a^^†^ 0.40–1.00	0.19 ^a^^†^ 0.09–0.46	0.42 ^a^* 0.39–0.54
Cd5 + Zn30	0.50 ^a^^†^ 0.40–0.70	0.20 ^a^* 0.13–0.27	0.23 ^a^^† c^* 0.15–0.30
Cd5 + Zn60	0.40 ^a^^‡ b^* ^c^* 0.20–1.00	0.13 ^a^^‡ b^* 0.05–0.22	0.08 ^a^^‡ b† c^^‡^ 0.04–0.17
Cd50	1.05 ^a^* 0.70–1.80	0.27 ^a^* 0.18–0.49	0.75 ^a^* ^f^^†^ 0.48–0.91
Cd50 + Zn30	0.40 ^a^^‡ b† c† g^* 0.20–0.50	0.16 ^a^^†^ 0.10–0.26	0.17 ^a^^‡ c†^ 0.09–0.22
Cd50 + Zn60	0.30 ^a^^‡ b† c† g^* 0.20–0.50	0.10 ^a^^‡ b‡^ 0.05–0.13	0.12 ^a^^‡ b† c‡ g^* 0.08–0.15

^2^ Data represent the median value and the minimum and maximum for 8 rats in each experimental group. Statistically significant differences (nonparametric Kruskal–Wallis test): ^a^ vs. Control group; ^b^ vs. Zn30 group; ^c^ vs. Zn60 group; ^f^ vs. Cd5 + Zn60 group; ^g^ vs. Cd50 group; * *p* < 0.05; ^†^ *p* < 0.01; ^‡^ *p* < 0.001. The directions and extent of differences between the experimental groups are presented in Supplementary Figure S7.

**Table 7:** The expression of ICAM-1 and L-selectin on leukocytes in the blood in particular experimental groups ^2^.

Experimental Group	ICAM-1 (%)		L-Selectin (%)	
	Median	Minimum and Maximum	Median	Minimum and Maximum
Control	0.114	0.038–0.300	28.4	23.7–31.4
Zn30	0.052	0.039–0.075	19.5	15.5–25.5
Zn60	0.051	0.039–0.059	34.8	28.0–41.9
Cd5	0.783	0.423–1.475	27.6	20.1–41.4
Cd5 + Zn30	2.295 ^a^^‡ b‡ c^^‡^	1.910–2.790	6.67 ^a^* ^c^^† d^*	4.20–8.97
Cd5 + Zn60	1.490 ^a^^† b† c†^	1.240–1.960	3.91 ^a^^† c^^‡ d†^	3.08–5.00
Cd50	0.329 ^e^*	0.064–0.441	22.1	12.6–28.3
Cd50 + Zn30	2.255 ^a^^‡ b‡ c^^‡ g^*	1.520–2.940	7.04 ^a^* ^c^^† g^*	6.36–9.00
Cd50 + Zn60	1.000	0.600–1.220	2.06 ^a^^‡ b^* ^c‡ d‡ g‡^	0.78–3.17

^2^ Data represent the median value and the minimum and maximum for 8 rats in each experimental group. Statistically significant differences (nonparametric Kruskal–Wallis test): ^a^ vs. Control group; ^b^ vs. Zn30 group; ^c^ vs. Zn60 group; ^d^ vs. Cd5 group; ^e^ vs. Cd5 + Zn30 group; ^g^ vs. Cd50 group; * *p* < 0.05; ^†^ *p* < 0.01; ^‡^ *p* < 0.001. The directions and extent of differences between the experimental groups are presented in Supplementary Figure S8.

**Table 8:** Mutual relationships between the investigated parameters in the vascular tissue of the abdominal aorta ^3^.

Parameter	Regression Analysis	TAS	TOS	OSI	TC	TG	eNOS	IL-1β	IL-10	PECAM-1	ICAM-1
TOS	β ^*p*^	NS									
	*R* ^2^										
OSI	β ^*p*^	–0.412 ^‡^	0.957 ^‡^								
	*R* ^2^	0.158	0.914								
TC	β ^*p*^	–0.423 ^‡^	0.665 ^‡^	0.757 ^‡^							
	*R* ^2^	0.167	0.435	0.566							
TG	β ^*p*^	–0.461 ^‡^	0.572 ^‡^	0.687 ^‡^	0.742 ^‡^						
	*R* ^2^	0.201	0.318	0.464	0.544						
eNOS	β ^*p*^	NS	0.773 ^‡^	0.751 ^‡^	0.510 ^‡^	0.480 ^‡^					
	*R* ^2^		0.592	0.558	0.250	0.220					
IL-1β	β ^*p*^	–0.625 ^‡^	0.566 ^‡^	0.664 ^‡^	0.672 ^‡^	0.513 ^‡^	0.468 ^‡^				
	*R* ^2^	0.381	0.311	0.432	0.444	0.253	0.208				
IL-10	β ^*p*^	NS	–0.420 ^‡^	–0.374 ^†^	NS	–0.242 *	–0.380 ^‡^	NS			
	*R* ^2^		0.164	0.127		0.045	0.131				
PECAM-1	β ^*p*^	0.526 ^‡^	NS	NS	–0.285 *	–0.224 ^#^	NS	–0.481 ^‡^	–0.504 ^‡^		
	*R* ^2^	0.266			0.068	0.036		0.220	0.243		
ICAM-1	β ^*p*^	0.528 ^‡^	NS	NS	–0.338 ^†^	–0.261 *	NS	–0.536 ^‡^	–0.420 ^‡^	0.965 ^‡^	
	*R* ^2^	0.268			0.101	0.055		0.277	0.164	0.931	
L-selectin	β ^*p*^	0.470 ^‡^	NS	NS	–0.270 *	NS	NS	–0.490 ^‡^	–0.489 ^‡^	0.966 ^‡^	0.954 ^‡^
	*R* ^2^	0.210			0.060			0.229	0.228	0.931	0.909

^3^ The results of the analysis of regression are presented as the β coefficient, *R*^2^_,_ and the level of statistical significance (^#^ p = 0.05; * *p* < 0.05; ^†^ *p* < 0.01; ^‡^ *p* < 0.001). NS, a lack of relationship (*p* > 0.05); TAS, total antioxidative status; TOS, total oxidative status; OSI, oxidative stress index; TC, total cholesterol; TG, triglycerides; eNOS, *endothelial nitric oxide synthase*; IL-1β, interleukin 1ꞵ; IL-10, interleukin 10; PECAM-1, platelet endothelial cell adhesion molecule-1; ICAM-1, intercellular adhesion molecule-1.

**Table 9:** Mutual relationships between the investigated parameters in the vascular tissue of the abdominal aorta and serum ^3^.

Serum	Regression Analysis	Vascular Tissue of the Abdominal Aorta										
		TAS	TOS	OSI	TC	TG	eNOS	IL-1β	IL-10	PECAM-1	ICAM-1	L-Selectin
CRP	β ^*p*^	–0.586 ^‡^	0.812 ^‡^	0.893 ^‡^	0.719 ^‡^	0.597 ^‡^	0.652 ^‡^	0.749 ^‡^	NS	–0.355 ^†^	–0.431 ^‡^	–0.334 ^†^
	*R* ^2^	0.334	0.654	0.794	0.510	0.347	0.416	0.555		0.113	0.174	0.099
IL-1β	β ^*p*^	–0.573 ^‡^	0.637 ^‡^	0.722 ^‡^	0.688 ^‡^	0.526 ^‡^	0.535 ^‡^	0.991 ^‡^	NS	–0.406 ^‡^	–0.474 ^‡^	–0.418 ^‡^
	*R* ^2^	0.319	0.397	0.514	0.466	0.267	0.276	0.982		0.153	0.213	0.163
IL-10	β ^*p*^	0.759 ^‡^	–0.368 ^†^	–0.497 ^‡^	–0.561 ^‡^	–0.495 ^‡^	–0.240 *	–0.760 ^‡^	–0.760 ^‡^	0.614 ^‡^	0.621 ^‡^	0.591 ^‡^
	*R* ^2^	0.569	0.123	0.236	0.305	0.234	0.044	0.571	0.571	0.368	0.377	0.340
VEGF	β ^*p*^	–0.245 *	0.476 ^‡^	0.481 ^‡^	0.581 ^‡^	0.547 ^‡^	0.317 ^†^	0.243 *	NS	NS	NS	NS
	*R* ^2^	0.046	0.215	0.220	0.328	0.289	0.088	0.046				

^3^ The results of the analysis of regression are presented as the β coefficient, *R*^2^, and the level of statistical significance (* *p* < 0.05; ^†^ *p* < 0.01; ^‡^ *p* < 0.001). NS, a lack of relationship (*p* > 0.05).

**Table 10:** Mutual relationships between the investigated parameters and the concentrations of Cd in the blood and Zn in the serum ^3^.

Biological Material	Regression Analysis	Cd in the Blood		Zn in the Serum	
		β ^*p*^	*R* ^2^	β ^*p*^	*R* ^2^
Vascular Tissue of the Abdominal Aorta	TAS	–0.428 ^‡^	0.171	0.375 ^†^	0.128
	TOS	0.443 ^‡^	0.185	–0.262 *	0.056
	OSI	0.488 ^‡^	0.227	–0.301 ^‡^	0.078
	TC	0.240 *	0.044	–0.328 ^†^	0.095
	TG	NS		–0.287 *	0.069
	eNOS	0.338 ^†^	0.101	NS	
	IL-1β	0.515 ^‡^	0.254	–0.310 ^†^	0.083
	IL-10	0.289 *	0,069	NS	
Endothelial Cells of the Abdominal Aorta	PECAM-1	–0.381 ^†^	0.133	0.403 ^‡^	0.150
	ICAM-1	–0.408 ^‡^	0.154	0.410 ^‡^	0.156
	L-selectin	–0.370 ^†^	0.124	0.350 ^†^	0.110
Blood Leukocytes	ICAM-1	NS		NS	
	L-selectin	–0.362 ^†^	0.118	NS	
Serum	IL-1β	0.495 ^‡^	0.234	–0.280 *	0.065
	IL-10	–0.613 ^‡^	0.367	0.462 ^‡^	0.202
	CRP	0.688 ^‡^	0.465	–0.431 ^‡^	0.174
	VEGF	NS		NS	

^3^ The results of the analysis of regression are presented as the β coefficient, *R*^2^, and the level of statistical significance. (* *p* < 0.05; ^†^ *p* < 0.01; ^‡^ *p* < 0.001). NS, a lack of relationship (*p* > 0.05).

## Data Availability

The data presented in this study are available on request from the corresponding authors. The data are not publicly available.

## Supplementary Materials

The following are available online at https://www.mdpi.com/article/10.3390/nu14194080/s1, Table S1: The concentration of Cd in the blood and urine in particular experimental groups, Table S2: The concentration of Cd in the blood and urine of inhabitants of industrialized countries, Table S3: The concentration of Zn in the serum and urine in particular experimental groups, Figure S1: The effect of Zn administration on TAS, TOS, and OSI in the vascular tissue of the abdominal aorta of rats intoxicated with Cd, Figure S2: The effect of Zn administration on the concentrations of TC and TG in the vascular tissue of the abdominal aorta of rats intoxicated with Cd, Figure S3: The effect of Zn administration on the concentration of eNOS in the vascular tissue of the abdominal aorta of rats intoxicated with Cd, Figure S4: The effect of Zn administration on the concentrations of IL-1β and IL-10 in the vascular tissue of the abdominal aorta and the serum of rats intoxicated with Cd, Figure S5: The effect of Zn administration on the concentration of CRP in the serum of rats intoxicated with Cd, Figure S6: The effect of Zn administration on the concentration of VEGF in the serum of rats intoxicated with Cd, Figure S7: The effect of Zn administration on the expression of PECAM-1, ICAM-1, and L-selectin on the endothelial cells of the abdominal aorta of rats intoxicated with Cd, Figure S8: The effect of Zn administration on the expression of ICAM-1 and L-selectin on the leukocytes in the blood of rats intoxicated with Cd. Refs [59,60,61,62,63,64,65,66,67,68,69] are cited in the Supplementary Materials.

## Abbreviations

b.w., body weight; Cd, cadmium; Cd^2+^, cadmium ion; CdCl_2_, cadmium chloride; CRP, C-reactive protein; CV, coefficient of variation; ELISA, enzyme-linked immunosorbent assay; eNOS, *endothelial nitric oxide synthase*; FR, free radicals; GSH, reduced glutathione; H_2_O_2_, hydrogen peroxide; ICAM-1, intercellular adhesion molecule-1; IL-1β, interleukin 1β; IL-10, interleukin 10; LDL-C, low-density lipoprotein cholesterol; NO, nitric oxide; OSI, oxidative stress index; PBS, physiological buffered saline; PECAM-1, platelet endothelial cell adhesion molecule-1; ROS, reactive oxygen species; SE, standard error; SD, standard deviation; SOD, superoxide dismutase; TAS, total antioxidative status; TC, total cholesterol; TG, triglycerides; TNF-α, *tumor necrosis factor α*; TOS, total oxidative status; VEGF, vascular endothelial growth factor; Zn, zinc; ZnCl_2_, zinc chloride; Zn^2+^, zinc ion.
