# Supplementary material for: The Beneficial Impact of Zinc Supplementation on the Vascular Tissue of the Abdominal Aorta under Repeated Intoxication with Cadmium: A Study in an In Vivo Experimental Model
Source: Nutrients. 2022 Sep 30;14(19):4080. doi: 10.3390/nu14194080 (PMC9570965; doi:10.3390/nu14194080)
Supplement: Supplementary file 1 [file nutrients-14-04080-s001.zip › nutrients-1917826-supplementary.pdf]

Supplementary Material

Supplementary Table S1. The concentration of Cd in the blood and urine in particular experimental groups.

Experimental Group	Cd in the Blood (μg/L)	Cd in the Urine (μg/24 h)
Control	0.284 ± 0.021 [1]	0.011 ± 0.001
Zn30	0.266 ± 0.021<br>↔	0.008 ± 0.001<br>↔
Zn60	0.309 ± 0.017<br>↔	0.011 ± 0.002<br>↔
Cd5	1.554 ± 0.121 [a‡ b‡ c‡]<br>↑ 5.5-fold	0.014 ± 0.001 [a* b†]<br>↑ 1.3-fold
Cd5 + Zn30	1.586 ± 0.050 [a‡ b‡]<br>↑ 5.5-fold	0.012 ± 0.002 [b*]<br>↔
Cd5 + Zn60	1.571 ± 0.053 [a‡ c‡]<br>↑ 5.5-fold	0.013 ± 0.002<br>↔
Cd50	15.50 ± 0.438 [a‡ b‡ c‡ d‡]<br>↑ 55-fold	0.226 ± 0.029 [a‡ b‡ c‡ d‡]<br>↑ 21.5-fold
Cd50 + Zn30	12.91 ± 0.289 [a‡ b‡ e‡ f‡]<br>↑ 45-fold	0.168 ± 0.025 [a‡ b‡ f‡]<br>↑ 16-fold
Cd50 + Zn60	13.27 ± 0.707 [a‡ c‡ e* g‡]<br>↑ 47-fold; ↘ *14%*	0.159 ± 0.021 [a‡ c‡ g‡]<br>↑ 15-fold; ↘ *30%*

[1] Data represent the mean ± standard error (SE) for 8 rats in each experimental group. Statistically significant differences: [a] vs. Control group; [b] vs. Zn30 group; [c] vs. Zn60 group; [d] vs. Cd5 group; [e] vs. Cd5 + Zn30 group; [f] vs. Cd5 + Zn60 group; [g] vs. Cd50 group; * $p < 0.05$; † $p < 0.01$; ‡ $p < 0.001$. ↑, ↔, an increase or lack of change, respectively, compared to the control group; ↘, a decrease compared to the Cd50 group. Detailed data on Cd concentration in all experimental groups have already been reported [1].

Supplementary Table S2. The concentration of Cd in the blood and urine of inhabitants of industrialized countries.

Country	Form of Cd concentration expression	Blood (μg Cd/L)		Urine (μg Cd/g creatinine)		Reference
		Female	Male	Female	Male	
Southern Korea	GM<br>(95% CI)	1.01<br>(0.99 – 1.03)	0.76<br>(0.74 – 0.77)			[2]
U.S.A.<br>tobacco smokers	GM<br>(95% CI)	1.17<br>(0.77 – 1.81)				[3]
South Korea<br>tobacco smokers	Mean (SD)	1.67 (0.68)				[4]
Southern Brasil	GM<br>(95% CI)<br>(Min, Max)	0.22<br>(0.19 – 0.25)<br>(0.004 – 3.77)	0.28<br>(0.24 – 0.32)<br>(0.004 – 3.08)			[5]
Taiwan	GM ± SD<br>(Min, Max)			0.55 ± 2.3<br>(ND – 7.56)	0.64 ± 2.3<br>(ND – 13.90)	[6]
Iran	Mean<br>(Min, Max)	0.59<br>(0.03 – 7.03)		0.51<br>(0.04 – 7.3)	0.32<br>(0.03 – 4.77)	[7]
Slovenia	GM<br>(95% CI)<br>(Min, Max)	0.35<br>(0.33 – 0.37)<br>(ND – 3.08)	0.23<br>(0.22 – 0.25)<br>(ND – 4.80)	0.17 μg/L<br>(0.16 – 0.19)<br>(ND – 3.83)	0.21 μg/L<br>(0.19 – 0.22)<br>(ND – 1.75)	[8]
Sweden<br>tobacco smokers	Median<br>(P5, P95)	1.00 (0.22 – 2.46)				[9]
China	Mean ± SD	0.36 ± 0.34	0.75 ± 0.81	1.94 ± 1.43	1.80 ± 1.52	[10]

Egypt occupational exposure	Mean ± SD		12.54 ± 4.20	8.21 ± 2.18	[11]
Czech Republic	GM (Range)	0.41 (0.11 – 2.1)			[12]
Poland	GM (Range)	0.65 (0.21 – 3.8)			
Slovakia	GM (Range)	0.40 (0.17 – 2.1)			
Slovenia	GM (Range)	0.49 (0.21 – 2.2)			
Sweden (north)	GM (Range)	0.25 (0.08 – 1.8)			
Sweden (south)	GM (Range)	0.35 (0.11 – 2.6)			
China	GM (Range)	0.99 (0.23 – 2.6)			
Ecuador	GM (Range)	0.61 (0.25 – 2.1)			
Morocco	GM (Range)	0.39 (0.15 – 1.8)			

GM, geometric mean; 95% CI, 95% confidence interval; Min, minimal value; Max, maximal value; ND, not detectable; SD, standard deviation; SE, standard error.

Supplementary Table S3. The concentration of Zn in the serum and urine in particular experimental groups.

Experimental Group	Zn in the Serum (μg/mL)	Zn in the Urine (μg/24 h)
Control	1.315 ± 0.037 [1]	2.877 ± 0.206
Zn30	1.255 ± 0.049<br>↔	3.114 ± 0.285<br>↔
Zn60	1.229 ± 0.057<br>↔	4.031 ± 0.343 ^{a*}<br>↑ 40%
Cd5	1.038 ± 0.055 $^{a*\ b*\ c*}$<br>↓ 21%	3.247 ± 0.244<br>↔
Cd5 + Zn30	1.188 ± 0.051<br>↔	3.057 ± 0.291<br>↔
Cd5 + Zn60	1.183 ± 0.048<br>↔	3.673 ± 0.290 ^{a*}<br>↑ 28%
Cd50	1.059 ± 0.067 $^{a\dagger\ b*}$<br>↓ 19%	3.790 ± 0.197 ^{a†}<br>↑ 32%
Cd50 + Zn30	1.063 ± 0.024 $^{a\ddagger\ b\dagger}$<br>↓ 19%	3.956 ± 0.398 ^{a*}<br>↑ 38%
Cd50 + Zn60	1.086 ± 0.068 $^{a*\ b*}$<br>↓ 17%	3.891 ± 0.383 ^{a*}<br>↑ 35%

[1] Data represent the mean ± standard error (SE) for 8 rats in each experimental group. Statistically significant differences: ^{a} vs. Control group; ^{b} vs. Zn30 group; ^{c} vs. Zn60 group; * $p < 0.05$; † $p < 0.01$; ‡ $p < 0.001$. ↑, ↓, ↔, an increase, decrease, or lack of change, respectively, compared to the control group. Detailed data on Zn concentration in all experimental groups have already been reported [1].

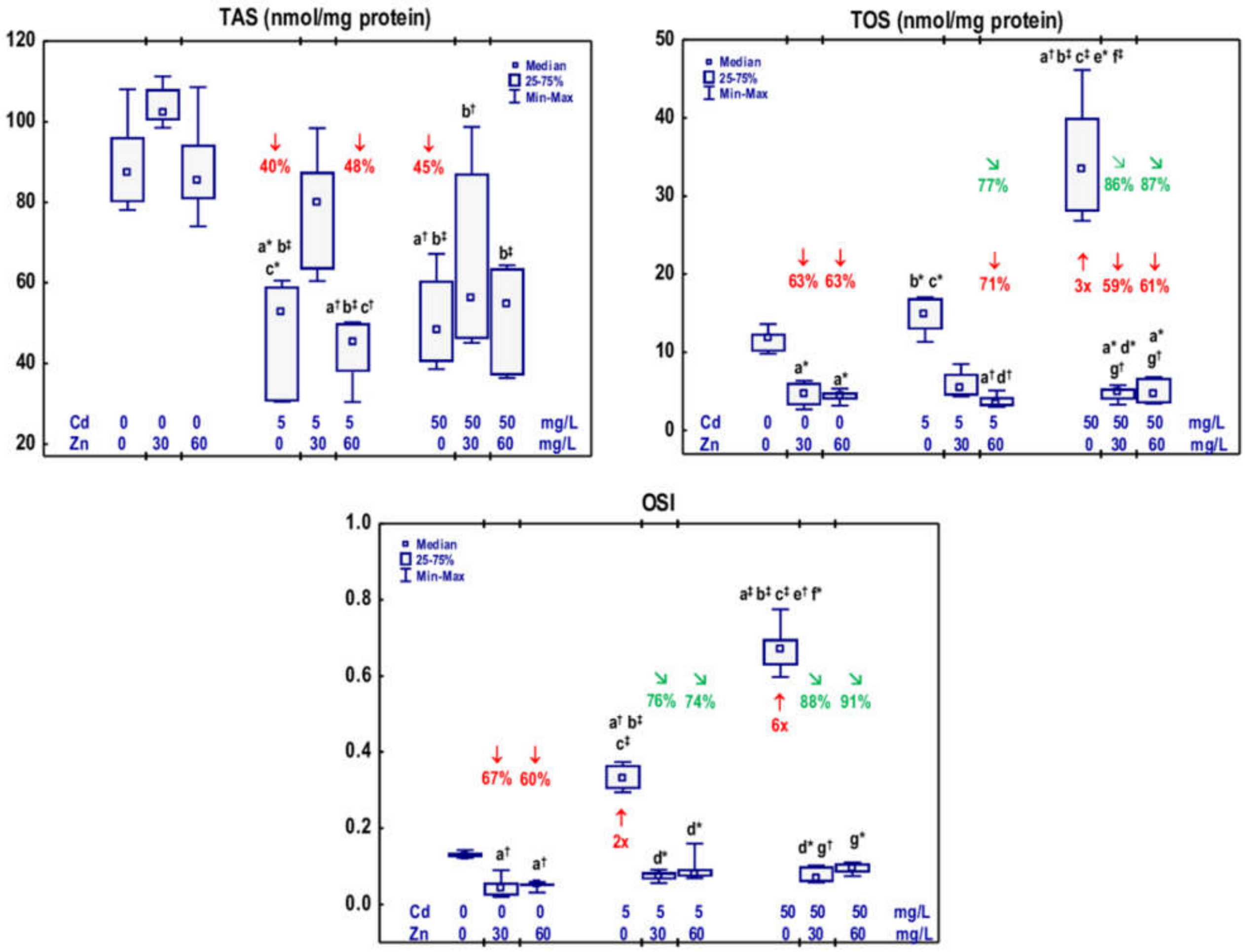

Supplementary Figure S1. The effect of Zn administration on TAS, TOS, and OSI in the vascular tissue of the abdominal aorta of rats intoxicated with Cd. The rats received 0, 5, and 50 mg Cd/L and/or 0, 30, and 60 mg Zn/L in drinking water for 6 months. Data are presented as median, 25–75% confidence interval, as well as the minimum (Min) and maximum (Max) values for 8 rats in each experimental group (numerical values of medians and minimum and maximum values are provided in the main text in Table 2). Statistically significant differences (nonparametric Kruskal–Wallis test): [a] vs. Control group; [b] vs. Zn30 group; [c] vs. Zn60 group; [d] vs. Cd5 group; [e] vs. Cd5 + Zn30 group; [f] vs. Cd5 + Zn60 group; [g] vs. Cd50 group; * $p < 0.05$; † $p < 0.01$; ‡ $p < 0.001$. Numerical values below or above the points presenting the median values disclose the percentage changes or factors of changes in comparison to the control group (↓, decrease; ↑, increase) or the respective group (Cd5 or Cd50) that did not receive this bioelement under the treatment with Cd (↘, decrease).

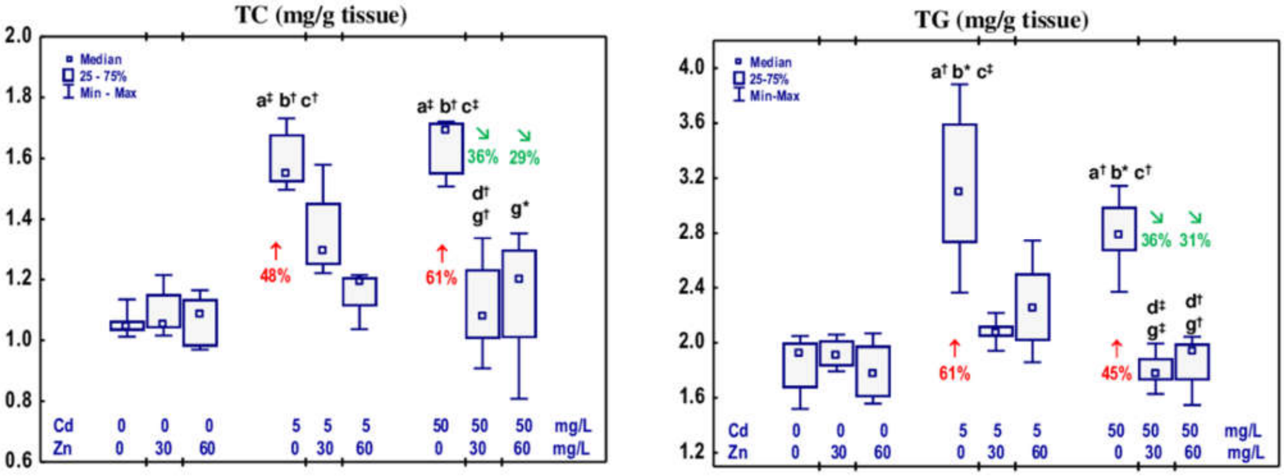

Figure S2. The effect of Zn administration on the concentrations of TC and TG in the vascular tissue of the abdominal aorta of rats intoxicated with Cd. The rats received 0, 5, and 50 mg Cd/L and/or 0, 30, and 60 mg Zn/L in drinking water for 6 months. Data are presented as median, 25–75% confidence interval, as well as the minimum (Min) and maximum (Max) values for 8 rats in each experimental group (numerical values of medians and minimum and maximum values are provided in the main text in Table 3). Statistically significant differences (nonparametric Kruskal–Wallis test): [a] vs. Control group; [b] vs. Zn30 group; [c] vs. Zn60 group; [d] vs. Cd5 group; [g] vs. Cd50 group; * $p < 0.05$; † $p < 0.01$; ‡ $p < 0.001$. Numerical values below or above the points presenting the median values disclose the percentage changes in comparison to the control group (↑, increase) or the Cd50 group (↘, decrease).

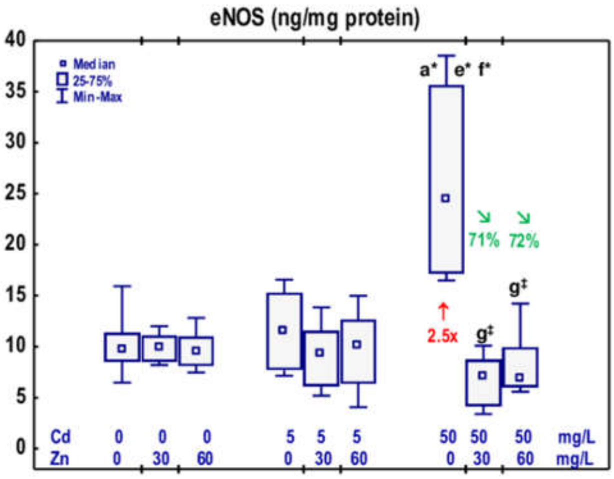

Supplementary Figure S3. The effect of Zn administration on the concentration of eNOS in the vascular tissue of the abdominal aorta of rats intoxicated with Cd. The rats received 0, 5, and 50 mg Cd/L and/or 0, 30, and 60 mg Zn/L in drinking water for 6 months. Data are presented as median, 25–75% confidence interval, as well as the minimum (Min) and maximum (Max) values for 8 rats in each experimental group (numerical values of medians and minimum and maximum values are provided in the main text in Table 3). Statistically significant differences (nonparametric Kruskal–Wallis test): [a] vs. Control group; [e] vs. Cd5 + Zn30 group; [f] vs. Cd5 + Zn60 group; [g] vs. Cd50 group; * $p < 0.05$; ‡ $p < 0.001$. Numerical values below or above the points presenting the median values disclose a factor of change or percentage changes, in comparison to the control group (↑, increase) or the Cd50 group (↘, decrease), respectively.

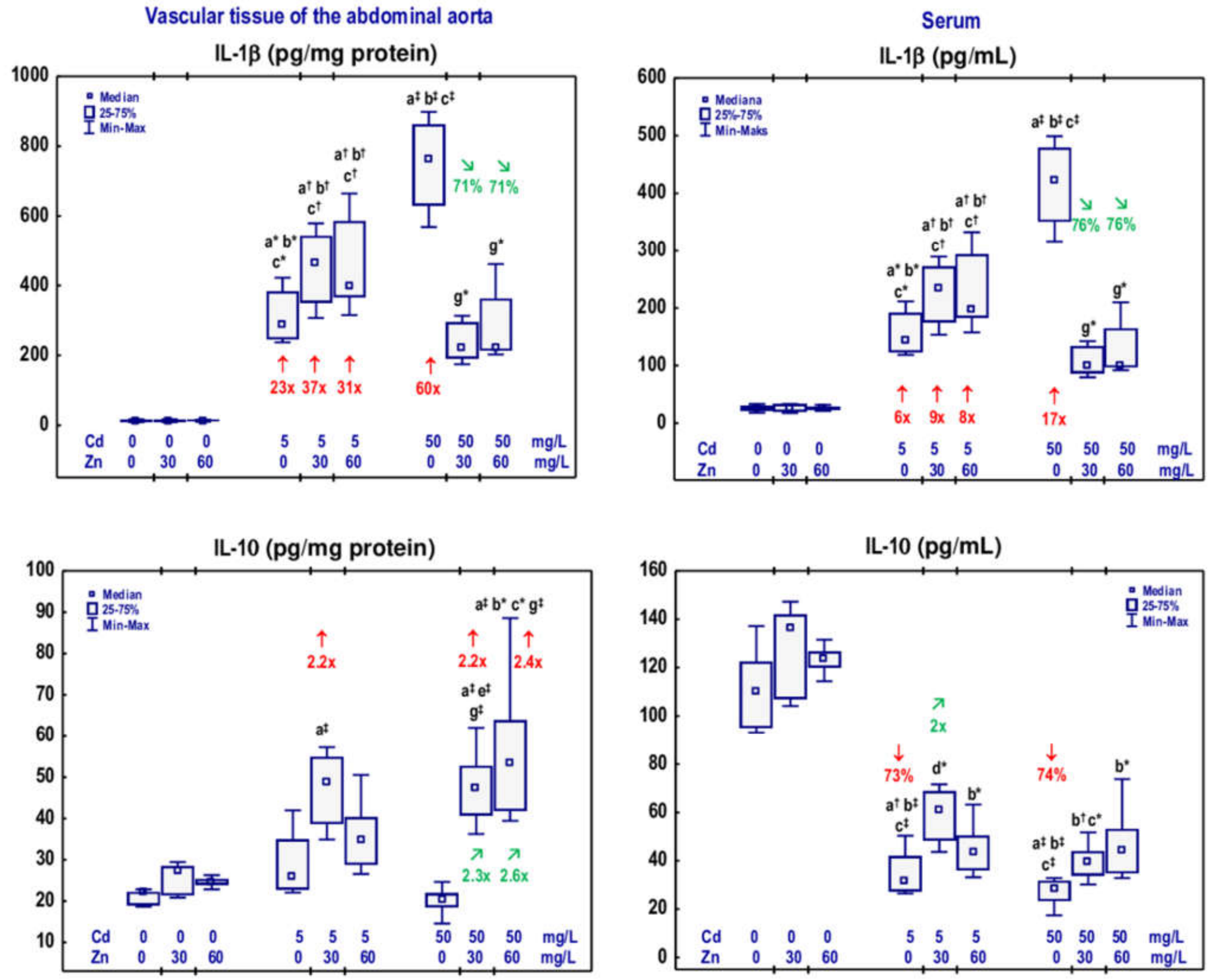

Supplementary Figure S4. The effect of Zn administration on the concentrations of IL-1β and IL-10 in the vascular tissue of the abdominal aorta and the serum of rats intoxicated with Cd. The rats received 0, 5, and 50 mg Cd/L and/or 0, 30, and 60 mg Zn/L in drinking water for 6 months. Data are presented as median, 25–75% confidence interval, as well as the minimum (Min) and maximum (Max) values for 8 rats in each experimental group (numerical values of medians and minimum and maximum values are provided in the main text in Table 4). Statistically significant differences (nonparametric Kruskal–Wallis test): [a] vs. Control group; [b] vs. Zn30 group; [c] vs. Zn60 group; [d] vs. Cd5 group; [e] vs. Cd5 + Zn30 group; [g] vs. Cd50 group; * $p < 0.05$; † $p < 0.01$; ‡ $p < 0.001$. Numerical values below or above the points presenting the median values disclose the percentage changes or factors of changes in comparison to the control group (↓, decrease; ↑, increase) or the respective group receiving Cd alone (↘, decrease; ↗, increase).

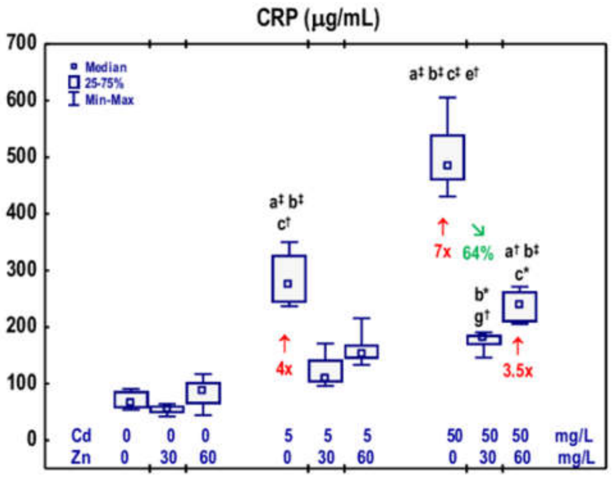

Supplementary Figure S5. The effect of Zn administration on the concentration of CRP in the serum of rats intoxicated with Cd. The rats received 0, 5, and 50 mg Cd/L and/or 0, 30, and 60 mg Zn/L in drinking water for 6 months. Data are presented as median, 25–75% confidence interval, as well as the minimum (Min) and maximum (Max) values for 8 rats in each experimental group (numerical values of medians and minimum and maximum values are provided in the main text in Table 4). Statistically significant differences (nonparametric Kruskal–Wallis test): [a] vs. Control group; [b] vs. Zn30 group; [c] vs. Zn60 group; [e] vs. Cd5 + Zn30 group; [g] vs. Cd50 group; * $p < 0.05$; † $p < 0.01$; ‡ $p < 0.001$. Numerical values below or above the points presenting the median values disclose the percentage changes or factors of changes in comparison to the control group (↑, increase) or the respective group receiving Cd alone (↘, decrease).

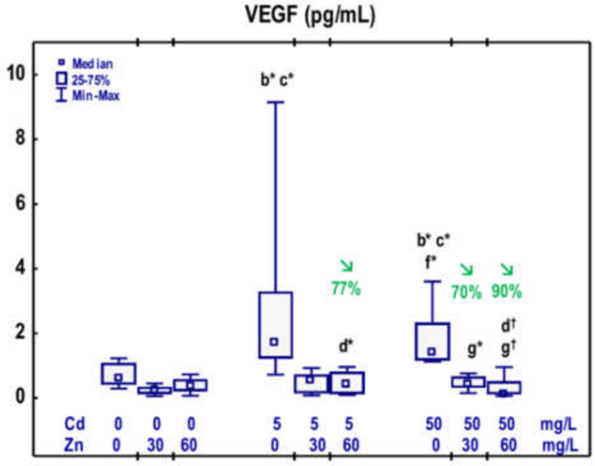

Supplementary Figure S6. The effect of Zn administration on the concentration of VEGF in the serum of rats intoxicated with Cd. The rats received 0, 5, and 50 mg Cd/L and/or 0, 30, and 60 mg Zn/L in drinking water for 6 months. Data are presented as median, 25–75% confidence interval, as well as the minimum (Min) and maximum (Max) values for 8 rats in each experimental group (numerical values of medians and minimum and maximum values are provided in the main text in Table 5). Statistically significant differences (nonparametric Kruskal–Wallis test): [b] vs. Zn30 group; [c] vs. Zn60 group; [d] vs. Cd5 group; [f] vs. Cd5 + Zn60 group; [g] vs. Cd50 group; * $p < 0.05$; † $p < 0.01$. Numerical values above the points presenting the median values disclose the percentage changes in comparison to the respective group receiving Cd alone (↘, decrease).

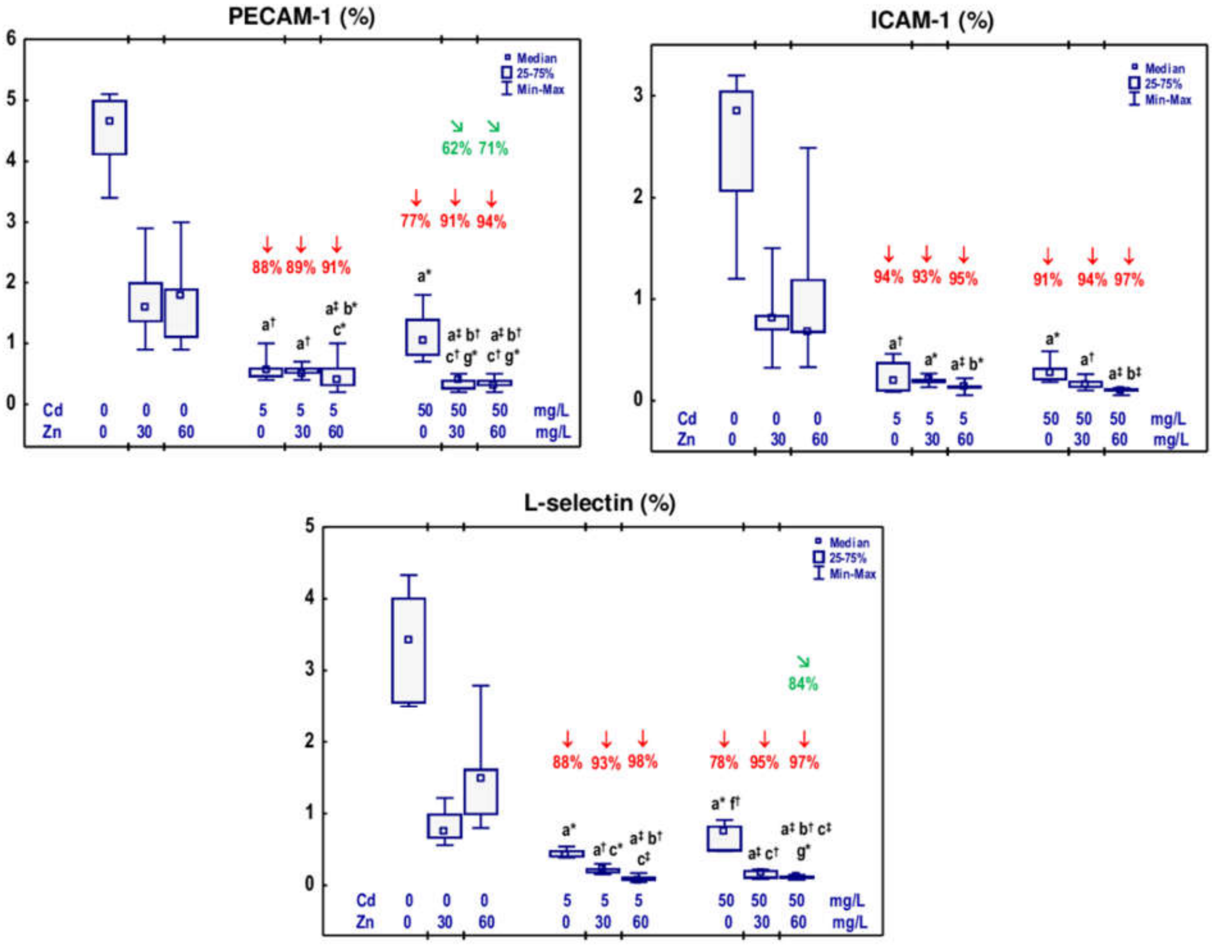

Supplementary Figure S7. The effect of Zn administration on the expression of PECAM-1, ICAM-1, and L-selectin on the endothelial cells of the abdominal aorta of rats intoxicated with Cd. The rats received 0, 5, and 50 mg Cd/L and/or 0, 30, and 60 mg Zn/L in drinking water for 6 months. Data are presented as median, 25–75% confidence interval, as well as the minimum (Min) and maximum (Max) values for 8 rats in each experimental group (numerical values of medians and minimum and maximum values are provided in the main text in Table 6). Statistically significant differences (nonparametric Kruskal–Wallis test): ^{a} vs. Control group; ^{b} vs. Zn30 group; ^{c} vs. Zn60 group; ^{f} vs. Cd5 + Zn60 group; ^{g} vs. Cd50 group; * $p < 0.05$; † $p < 0.01$; ‡ $p < 0.001$. Numerical values above the points presenting the median values disclose the percentage changes in comparison to the control group (↓, decrease) or the Cd50 group (↘, decrease).

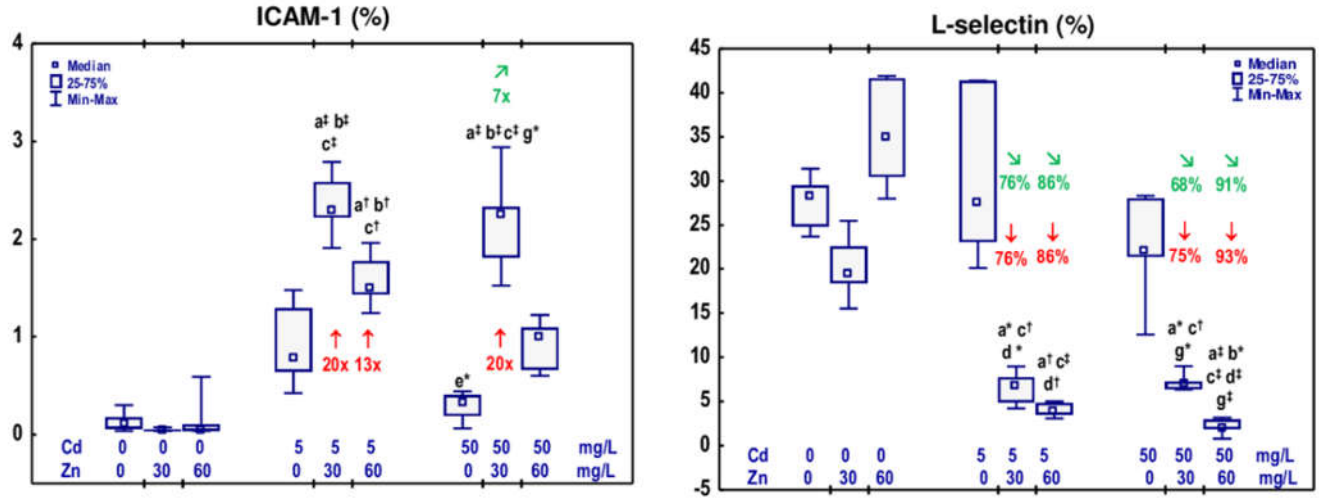

Supplementary Figure S8. The effect of Zn administration on the expression of ICAM-1 and L-selectin on the leukocytes in the blood of rats intoxicated with Cd. The rats received 0, 5, and 50 mg Cd/L and/or 0, 30, and 60 mg Zn/L in drinking water for 6 months. Data are presented as median, 25–75% confidence interval, as well as the minimum (Min) and maximum (Max) values for 8 rats in each experimental group (numerical values of medians and minimum and maximum values are provided in the main text in Table 7). Statistically significant differences (nonparametric Kruskal–Wallis test): ^{a} vs. Control group; ^{b} vs. Zn30 group; ^{c} vs. Zn60 group; ^{d} vs. Cd5 group; ^{e} vs. Cd5 + Zn30 group; ^{g} vs. Cd50 group; * $p < 0.05$; † $p < 0.01$; ‡ $p < 0.001$. Numerical values below or above the points presenting the median values disclose the percentage changes or factors of changes in comparison to the control group (↓, decrease; ↑, increase) or the respective group receiving Cd alone (↘, decrease; ↗, increase).

References

1. Brzóska, M.M.; Rogalska, J.; Gałażyn-Sidorczuk, M.; Jurczuk, M.; Roszczenko, A.; Kulikowska-Karpińska, E.; Moniuszko-Jakoniuk, J. Effect of zinc supplementation on bone metabolism in male rats chronically exposed to cadmium. Toxicology **2007**, *237*, 89–103. doi: 10.1016/j.tox.2007.05.001.
2. Park, E.; Kim, J.; Kim, B.; Park, E.Y. Association between environmental exposure to cadmium and risk of suspected non-alcoholic fatty liver disease. *Chemosphere* **2021**, *246*, 128947. https://doi.org/10.1016/j.chemosphere.2020.128947.
3. Domingo-Relloso, A.; Riffo-Campos, A.L.; Haack, K.; Rentero-Garrido, P.; Ladd-Acosta, C.; Fallin, D.M.; Tang, W.Y.; Herreros-Martinez, M.; Gonzalez, J.R.; Bozack, A.K.; Cole, S.A.; Navas-Acien, A.; Tellez-Plaza, M. Cadmium, smoking, and human blood DNA methylation profiles in adults from the strong heart study. *Environ. Health Perspect.* **2020**, *128*, 1–14. https://doi.org/10.1289/EHP6345.
4. Lee, J.E.; Kim, H.R.; Lee, M.; Kim, N.H.; Wang, K.M.; Lee, S.; Park, O.; Hong, E.J.; Youn, J.W.; Kim, Y.Y. Smoking-related DNA methylation is differentially associated with cadmium concentration in blood. *Biochem. Genetics*, **2020,** *58*, 617–630. https://doi.org/10.1007/s10528-020-09965-y.
5. Martins, A.C.; Urbano, M.R.; Lopes, A.C.B.A.; Carvalho, M.F.H.; Buzzo, M.L.; Docea, A.O.; Mesas, A.E.; Aschner, M.; Silva, A.M.R.; Silbergeld, E.K.; Paoliello, M.M.B. Blood cadmium levels and sources of exposure in an adult urban population in southern Brazil. *Environ. Res.* **2020**, *187*, 109618. doi.org/10.1016/j.envres.2020.109618.
6. Liao, K.W.; Pan, W.H.; Liou, S.H.; Sun, C.W.; Huang, P.C.; Wang, S.L. Levels and temporal variations of urinary lead, cadmium, cobalt, and copper exposure in the general population of Taiwan. *Environ. Sci. Pollut. Res. Int*. **2019**, *26*, 6048–6064. doi.org/10.1007/s11356-018-3911-0.
7. Ghoochani, M.; Dehghani, M.H.; Rastkari, N.; Nodehi, R.N.; Yunesian, M.; Mesdaghinia, A.; Houshiarrad, A.; Saraei, M. Association among sources exposure of cadmium in the adult non-smoking general population of Tehran. *Biol. Trace. Elem. Res.* **2019**, *191*, 27–33. https://doi.org/10.1007/s12011-018-1590-9.
8. Tratnik, J.S.; Falnoga, I.; Mazej, D.; Kocman, D.; Fajon, V.; Jagodic, M.; Stajnko, A.; Trdin, A.; Šlejkovec, Z.; Jeran, Z.; Osredkar, J.; Sešek-Briški, A.; Krsnik, M.; Kobal, A.B.; Kononenko, L.; Horvat, M. Results of the first national human biomonitoring in Slovenia: Trace elements in men and lactating women, predictors of exposure and reference values. *Int. J. Hyg. Environ. Health* **2019**, *222*, 563–582. https://doi.org/10.1016/j.ijheh.2019.02.008.
9. Li, H.; Fagerberg, B.; Sallsten, G.; Borné, Y.; Hedblad, B.; Engström, G.; Barregard, L.; Andersson, E.M. Smoking-induced risk of future cardiovascular disease is partly mediated by cadmium in tobacco: Malmö Diet and Cancer Cohort Study. *Environ. Health*, **2019**, *18*, 56-66. https://doi.org/10.1186/s12940-019-0495-1.
10. Chen, X.; Zhu, G.; Wang, Z.; Liang, Y.; Chen, B.; He, P.; Nordberg, M.; Nordberg, G. F.; Ding, X.; Jin, T. The association between dietary cadmium exposure and renal dysfunction – the benchmark dose estimation of reference levels: the ChinaCad study. *J. Appl. Toxicol.* **2018**, *38*, 1365–1373. https://doi.org/10.1002/jat.3647.
11. Mortada, W.I.; Hassanien, M.M.; Donia, A.F.; Shokeir, A.A. Application of cloud point extraction for cadmium in biological samples of occupationally exposed workers: relation between cadmium exposure and renal lesion. *Biol. Trace Elem. Res.* **2015**, *168*, 303-310. doi: 10.1007/s12011-015-0365-9.
12. Pawlas, N.; Strömberg, U.; Carlberg, B.; Cerna, M.; Harari, F.; Harari, R.; Horvat, M., Hruba, F.; Koppova, K.; Krskova, A.; Krsnik, M.; Li, Y.F.; Löfmark, L.; Lundh, T.; Lundström, N.G.; Lyoussi, B.; Markiewicz-Górka, I.; Mazej, D.; Osredkar, J.; … Bergdahl, I. A. Cadmium, mercury and lead in the blood of urban women in Croatia, the Czech Republic, Poland, Slovakia, Slovenia, Sweden, China, Ecuador and Morocco. *Int. J. Occup. Med. Environ. Health* **2013**, *26*, 58–72. https://doi.org/10.2478/S13382-013-0071-9.
